# Serum Argininosuccinic Acid Is Associated with Diabetes in Patients with Coronary Artery Disease: An Exploratory Targeted LC–MS/MS Study

**DOI:** 10.3390/ijms27198766

**Published:** 2026-09-30

**Authors:** Beata Krasińska, Minseung Kang, Siwon Baek, Dagmara Pietkiewicz, Szymon Plewa, Mariusz Kowalewski, Giuseppe Maria Raffa, Calogera Pisano, Piotr Suwalski, Ievgen Spasenenko, Katarzyna Gabriel, Łukasz Stryczyński, Anna Olasińska-Wiśniewska, Jan Matysiak, Zbigniew Krasiński, Andrzej Tykarski, Tomasz Urbanowicz

**Affiliations:** 1 Department of Hypertensiology, Angiology, and Internal Medicine, Poznan University of Medical Sciences, ½ Dluga Street, 61-848 Poznan, Poland; 2 College of Medicine, Korea University, 73 Goryeodae-ro, Seongbuk-gu, Seoul 02841, Republic of Korea; 3 College of Medicine, Inje University, 75 Bokji-ro, Busanjin-gu, Busan 47392, Republic of Korea; 4 Centre for Innovative Pharmaceutical Technology, Poznan University of Medical Sciences, Rokietnicka 3, 60-806 Poznan, Poland; 5 Department of Inorganic and Analytical Chemistry, Faculty of Pharmacy, Poznan University of Medical Sciences, 61-614 Poznan, Poland; 6 Thoracic Research Center, Collegium Medicum, Nicolaus Copernicus University, 85-094 Bydgoszcz, Poland; 7 Department of Precision Medicine in Medical Surgical and Critical Area (Me.Pre.C.C.), University of Palermo, 90134 Palermo, Italy; 8 Department of Cardiac Surgery, Center of Postgraduate Medical Education, Central Clinical Hospital of the Ministry of Interior, 02-507 Warszawa, Poland; 9 Department of Research, IRCCS ISMETT, 90127 Palermo, Italy; 10 Cardiac Surgery and Transplantology Department, Poznan University of Medical Sciences, ½ Długa, 61-848 Poznan, Poland; 11 Department of Vascular, Endovascular Surgery, Angiology and Phlebology, Poznan University of Medical Sciences, ½ Dluga Street, 61-848 Poznan, Poland

**Keywords:** argininosuccinic acid, diabetes mellitus, coronary artery disease, targeted metabolomics, LC–MS/MS, renal function, arginine metabolism

## Abstract

Diabetes mellitus is associated with metabolic alterations, but amino-acid differences among patients with coronary artery disease (CAD) remain incompletely characterized. We screened 45 amino acids and related metabolites by diabetes status in an exploratory retrospective disease-restricted cohort (recorded diseased-vessel count ≥ 1; *n* = 33). Argininosuccinic acid was available for 26 patients. Comparisons used exact Wilcoxon tests, Cliff’s delta, and Benjamini–Hochberg correction. Estimated argininosuccinic acid concentrations were higher in diabetes (0.28 [0.21–0.34] vs. 0.15 [0.13–0.19] µmol/L; Cliff’s delta = 0.765; *p* < 0.001; q = 0.035), the only association surviving false-discovery-rate correction. All argininosuccinic acid estimates were extrapolated below the lowest calibrator and should be interpreted as semiquantitative. The diabetes coefficient remained positive in crude and age- and sex-adjusted HC3-robust models, but renal and influence analyses were imprecise. Argininosuccinic acid correlated inversely with eGFR (rho = −0.445; *p* = 0.023) and positively with creatinine (rho = 0.486; *p* = 0.012). The diabetes-by-CAD interaction was not significant (*p* = 0.183). These data suggest a hypothesis-generating association requiring validation with an appropriate calibration range and careful renal phenotyping.

## 1. Introduction

Diabetes mellitus is a major determinant of cardiovascular morbidity and mortality and confers approximately twice the risk of coronary heart disease observed in individuals without diabetes, even after conventional vascular risk factors are considered [1]. Among patients with established coronary artery disease (CAD), diabetes is associated with diffuse and accelerated atherosclerosis, endothelial dysfunction, chronic inflammation, dyslipidemia, renal impairment, and adverse cardiovascular outcomes. Nevertheless, the biological phenotype of diabetes within CAD is heterogeneous, and routinely measured clinical variables suggest that important molecular determinants of diabetes-associated coronary disease remain unidentified despite conventional clinical phenotyping.

Metabolomic profiling provides a complementary approach because circulating small molecules integrate genetic background, nutritional exposure, medication effects, organ function, and active disease biology [2]. Prospective studies have repeatedly implicated branched-chain and aromatic amino acids in the development of type 2 diabetes [3], and a diabetes-predictive amino-acid score has also been associated with subsequent cardiovascular events [4]. Population-based cardiovascular studies have linked phenylalanine and other metabolic measures to incident cardiovascular disease, and higher circulating levels of branched-chain amino acids have been associated with future cardiovascular risk [5,6]. In individuals with established type 2 diabetes, however, associations between individual amino acids and macrovascular outcomes have been less uniform after adjustment for adiposity, renal function, glycemic control, and conventional cardiovascular risk factors [7]. Despite these observations, no reproducible metabolomic signature has emerged to specifically characterize diabetes in established coronary artery disease.

Studies focused specifically on the coexistence of diabetes and CAD have likewise identified heterogeneous metabolic signatures. Quantitative nuclear magnetic resonance profiling demonstrated differences in branched-chain and aromatic amino acid metabolism between patients with coronary heart disease, with and without type 2 diabetes [8]. Untargeted metabolomics in patients with type 2 diabetes and CAD implicated disturbances in branched-chain amino acids, glucose, and lipid metabolism [9], whereas a capillary electrophoresis–mass spectrometry pilot study identified a distinct panel of metabolites associated with subsequent CAD in patients with diabetes [10]. Similarly, a targeted case–control study reported differences in circulating serine, acylcarnitine, and phospholipid metabolites according to CAD status among patients with type 2 diabetes [11]. More recently, comparative metabolomic analyses of patients with CAD with and without type 2 diabetes further suggested that diabetes is associated with additional alterations in lipid and intermediary metabolism within CAD [12]. Collectively, these findings suggest that CAD accompanied by diabetes is associated with a distinct, but heterogeneous metabolic profile. The variation across studies indicates that analytical platform, population characteristics, disease stage, renal function, and treatment exposure may strongly influence the metabolites identified. Arginine recycling is increasingly recognized as a dynamic regulator of endothelial nitric oxide production rather than merely a component of intermediary amino acid metabolism.

Arginine-related metabolism provides a biologically plausible framework for interpreting argininosuccinic acid, but circulating concentrations cannot identify the responsible tissue or enzymatic step. Endothelial nitric oxide synthase (eNOS) generates nitric oxide (NO) from arginine, and arginine can be regenerated from citrulline through argininosuccinate synthase 1 (ASS1) and argininosuccinate lyase (ASL) [13,14,15,16,17]. Experimental disruption of this recycling system can impair NO production [18,19], and diabetes has been associated with oxidative stress, altered insulin signaling, and changes in arginine-related pathways [20,21,22,23,24,25]. However, argininosuccinic acid also participates in systemic amino-acid and urea-cycle metabolism, and its circulating level may reflect renal handling, hepatic metabolism, diet, medication exposure, or other tissue-level processes. Accordingly, an endothelial arginine–NO interpretation remains mechanistic context rather than a direct measurement in the present study.

Accordingly, this exploratory study evaluated whether circulating amino acids and related metabolites differed by diabetes status in patients with documented coronary artery disease. The disease-restricted metabolite screen was used to identify candidate signals for follow-up analysis, after which the leading metabolite was evaluated with demographic, renal, transformation, and influence sensitivity analyses. Secondary analyses examined the full metabolic cohort, missingness, renal associations, and a formal diabetes-by-CAD interaction in participants with angiographic data. Because the study did not measure NO production, endothelial function, ASS1/ASL activity, arginase activity, or metabolic flux, mechanistic interpretations were treated as hypotheses requiring independent validation.

**Figure 1:**
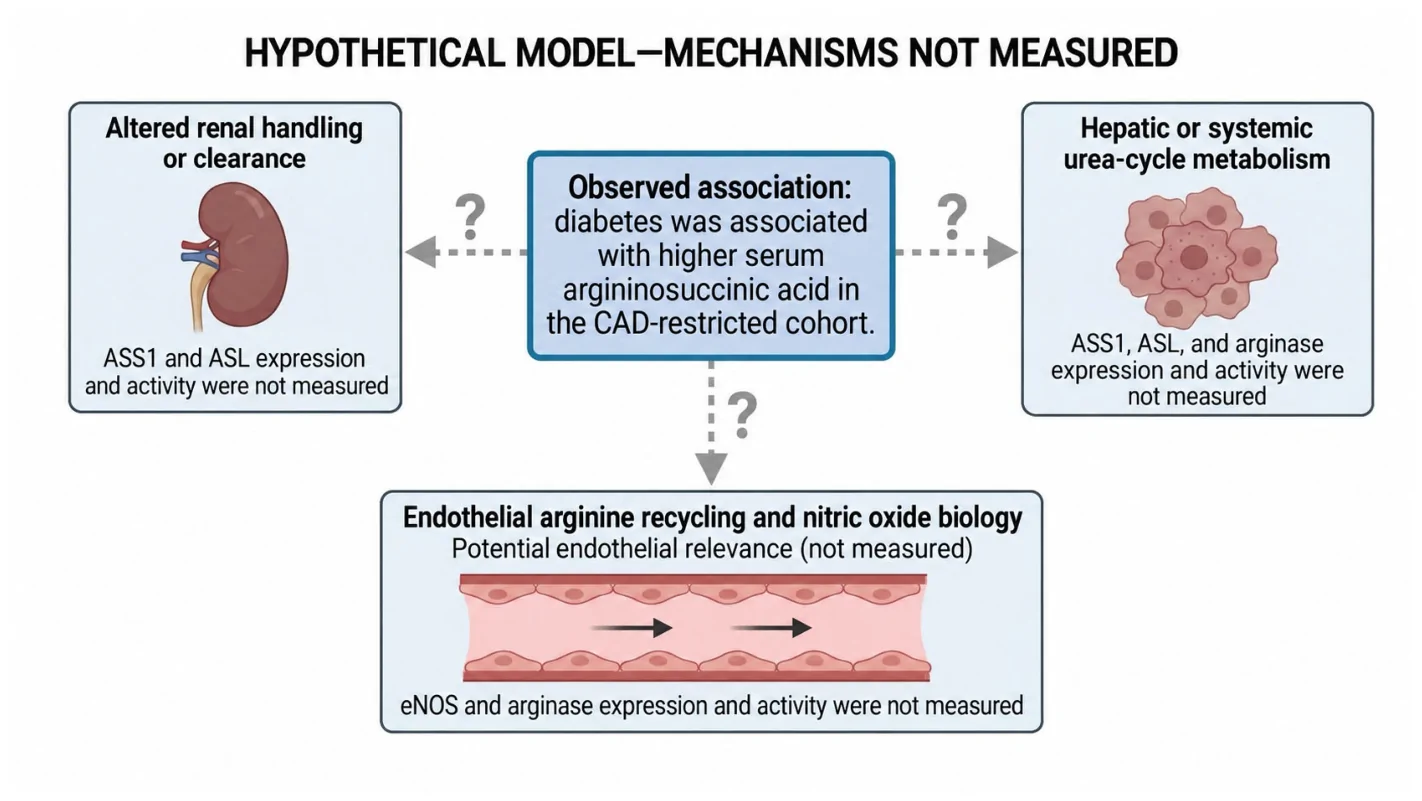
Hypothetical framework relating the observed association between diabetes and higher serum argininosuccinic acid to possible renal, hepatic or systemic, and endothelial explanations. These mechanisms and the depicted enzyme expression and activity were not measured in the present study.

Figure 1 presents a hypothetical biological framework linking diabetes-associated metabolic stress, arginine recycling, and endothelial NO biology. The figure is intended only to provide context for the measured serum metabolite association and should not be interpreted as evidence that any depicted enzymatic step was altered in the study participants.

## 2. Results

### 2.1. Study Population and Clinical Characteristics

After consolidating repeated source records, the participant-level database comprised 97 patients. Argininosuccinic acid estimates were available for 73 of 97 participants (50 without diabetes and 23 with diabetes). Coronary vessel count was available for 70 patients, of whom 33 had at least one recorded diseased coronary vessel and constituted the disease-restricted analysis cohort. This cohort included 21 patients without diabetes and 12 with diabetes. The groups differed in several laboratory characteristics, including higher white blood cell and neutrophil counts, higher serum glucose and HbA1c, lower HDL cholesterol, and higher triglycerides in the diabetes group. Age, sex, BMI, and recalculated continuous 2021 CKD-EPI eGFR did not differ statistically between groups. Because several clinical variables were incompletely recorded, group-specific denominators are reported in Table 1.

Continuous variables are presented as median [interquartile range] with the number of available observations shown in each group; *p*-values were calculated using two-sided exact Wilcoxon rank-sum tests. Binary variables are presented as n/N (%) and were compared using Fisher’s exact test. eGFR values are continuous estimates recalculated with the 2021 race-free CKD-EPI creatinine equation. Smoking status was sparsely recorded (5/33) and is presented descriptively without inferential testing.

Given the observed clinical and laboratory differences and the incomplete recording of several variables, the subsequent metabolomic findings were interpreted as exploratory associations rather than as evidence from clinically equivalent groups.

### 2.2. Diabetes-Associated Amino-Acid Profile

**Figure 2:**
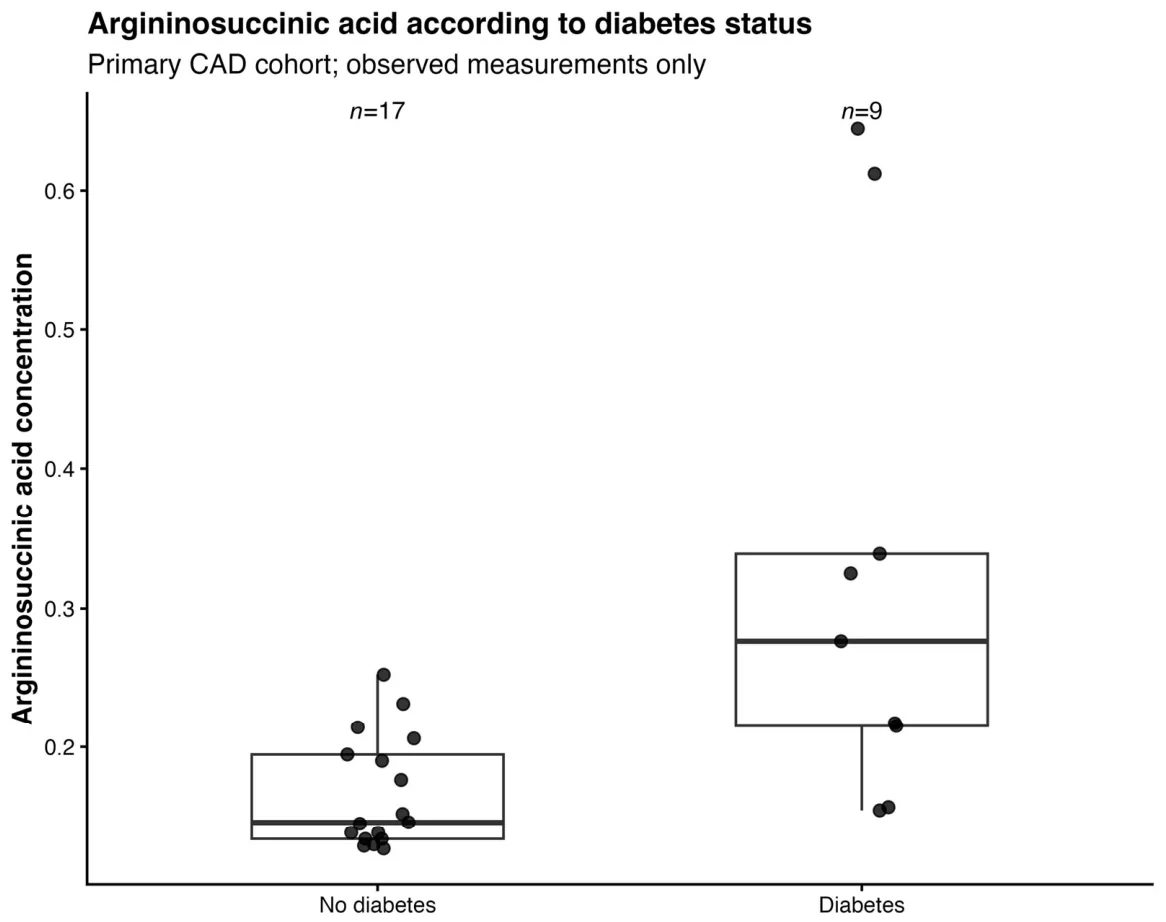
Estimated argininosuccinic acid concentrations, extrapolated below the lowest calibrator (2.64 µmol/L), according to diabetes status in the disease-restricted cohort (*n* = 17 without diabetes; *n* = 9 with diabetes). Boxes show the interquartile range with the median; whiskers extend to the most extreme observation within 1.5× the interquartile range, and points represent participant-level estimates. This comparison is semiquantitative.

Within the disease-restricted metabolite screen, argininosuccinic acid had the largest diabetes-associated effect among metabolites with sufficient observations and was the only metabolite significant after FDR correction across 39 valid comparisons. Argininosuccinic acid estimates were available for 26 of 33 participants (17 of 21 without diabetes and 9 of 12 with diabetes). Seven values were missing and were not imputed; the reasons were unknown in the available analytic records. All 26 estimates exceeded the signal-to-noise-derived estimated LOQ of 0.07368 µmol/L, but all fell below the lowest calibrator (2.64 µmol/L) and therefore constitute semiquantitative extrapolations. The median estimated concentration was 0.28 [0.21–0.34] µmol/L with diabetes and 0.15 [0.13–0.19] µmol/L without diabetes (Cliff’s delta = 0.765; exact *p* < 0.001; FDR q = 0.035; Table 2 and Figure 2).

**Figure 3:**
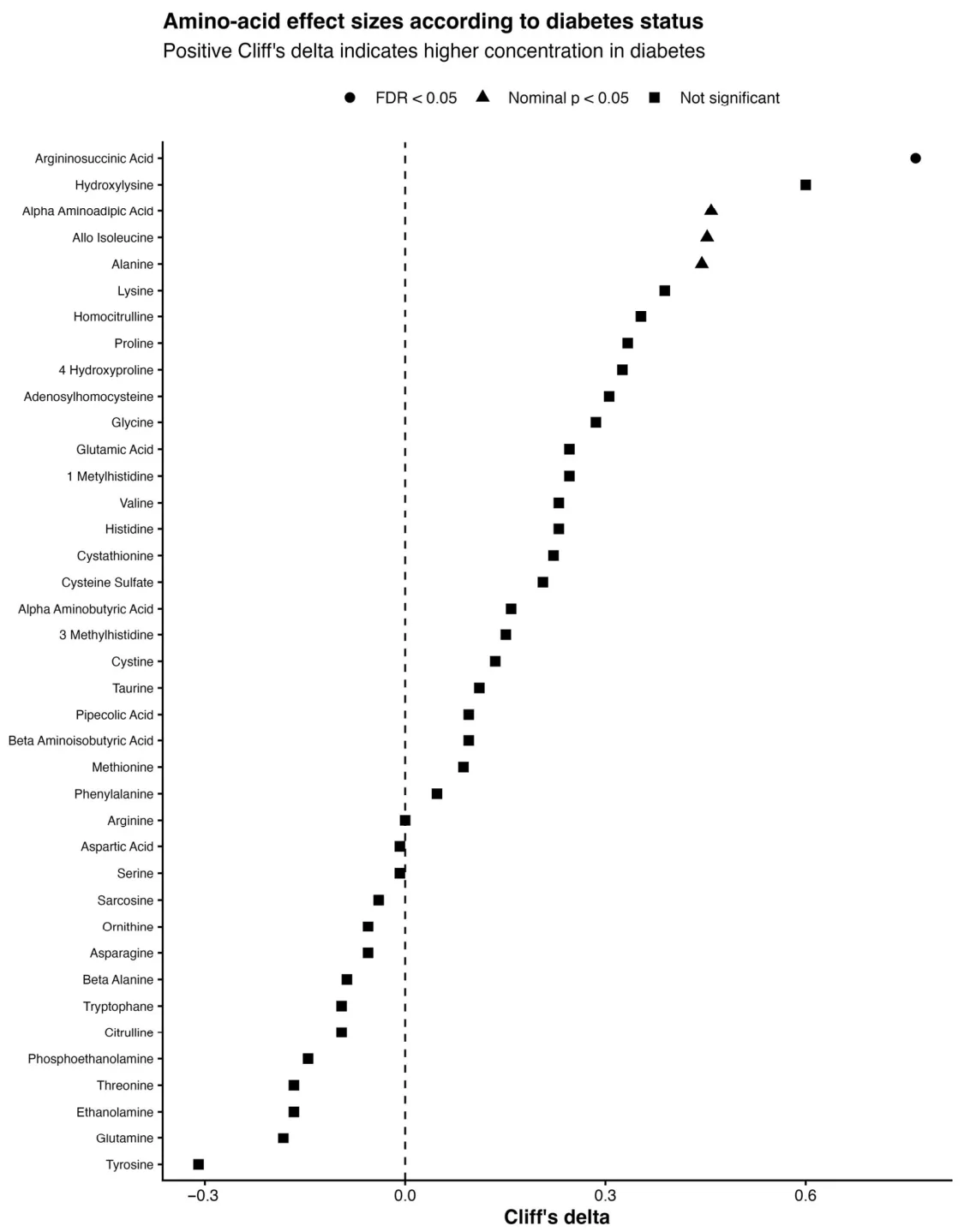
Amino-acid effect sizes according to diabetes status in the disease-restricted cohort. Positive Cliff’s delta indicates higher concentrations in patients with diabetes. Symbols distinguish the FDR-significant finding from metabolites meeting only nominal *p* < 0.05 and from nonsignificant results. The argininosuccinic acid effect size is based on extrapolated, semiquantitative estimates.

Alpha-aminoadipic acid, alloisoleucine, and alanine showed nominal differences but did not remain significant after multiplicity correction (Figure 3).

Accordingly, argininosuccinic acid was treated as the leading exploratory candidate for adjusted and sensitivity analyses; this result does not by itself establish a CAD-specific effect or a specific biochemical mechanism.

Values are median [interquartile range] with group-specific available *n*. A positive Cliff’s delta indicates a higher concentration among patients with diabetes. Exact *p*-values were adjusted using the Benjamini–Hochberg procedure within the disease-restricted metabolite screen. FDR correction covered 39 metabolites with valid two-group tests; the argininosuccinic acid values are estimated concentrations extrapolated below the lowest calibrator (2.64 µmol/L).

### 2.3. Adjusted and Sensitivity Analyses

**Figure 4:**
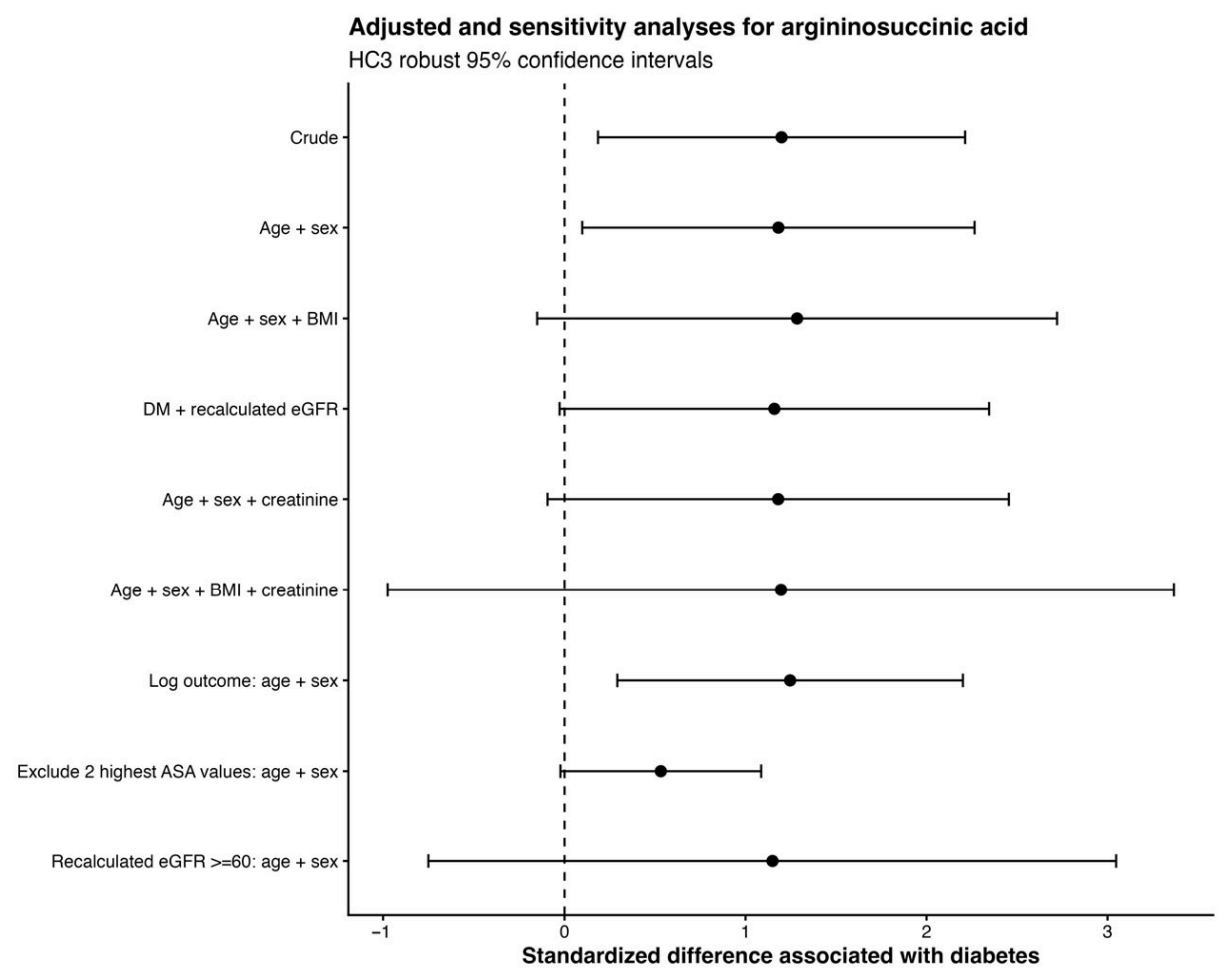
HC3-robust adjusted and sensitivity analyses for argininosuccinic acid. Points represent standardized diabetes coefficients and horizontal bars represent 95% confidence intervals. Sample sizes vary according to covariate availability.

Using HC3 heteroscedasticity-consistent standard errors, diabetes was positively associated with standardized argininosuccinic acid in the crude model (β = 1.199 SD units; 95% CI, 0.185 to 2.213; *p* = 0.022) and after adjustment for age and sex (β = 1.182; 95% CI, 0.098 to 2.265; *p* = 0.034). The direction remained positive after additional adjustment or sensitivity analysis, but confidence intervals crossed zero in several models (Table 3 and Figure 4).

Adjustment for recalculated continuous eGFR yielded β = 1.159 (95% CI, −0.027 to 2.346; *p* = 0.055), and the age-, sex-, and creatinine-adjusted model yielded β = 1.180 (95% CI, −0.094 to 2.455; *p* = 0.068). The age-, sex-, and BMI-adjusted model was also imprecise (*n* = 19; *p* = 0.076), reflecting the limited complete-case sample. The log-transformed and subsequently standardized outcome remained positively associated with diabetes after age and sex adjustment (β = 1.246; 95% CI, 0.292 to 2.201; *p* = 0.013).

Exclusion of the two highest argininosuccinic acid concentrations attenuated the age- and sex-adjusted estimate (β = 0.532; 95% CI, −0.022 to 1.086; *p* = 0.059). Restriction to participants with recalculated eGFR ≥ 60 mL/min/1.73 m^2^ was similarly imprecise (*n* = 20; *p* = 0.218). These analyses indicate a consistently positive effect direction but also substantial uncertainty related to sample size, renal adjustment, and influential observations.

The sensitivity analyses therefore support persistence of a positive association direction while showing that statistical precision is limited and that renal independence cannot be established in this dataset.

### 2.4. Secondary and Supplementary Analyses

In the full metabolic cohort, ethanolamine was lower in diabetes and was the only metabolite that remained significant after FDR correction (q = 0.048), whereas argininosuccinic acid was higher only at the nominal level (*p* = 0.008; q = 0.129). This difference demonstrates that the identity of the leading metabolite depended on the analysis population, but it does not establish effect specificity to CAD.

A formal diabetes-by-CAD interaction analysis among participants with available argininosuccinic acid and angiographic disease status (*n* = 52) did not show statistically significant effect modification by recorded CAD status (crude interaction *p* = 0.167; age- and sex-adjusted *p* = 0.183; log-outcome age- and sex-adjusted *p* = 0.210). Argininosuccinic acid correlated inversely with recalculated continuous 2021 CKD-EPI eGFR in the pooled disease-restricted sample (rho = −0.445; *p* = 0.023) and positively with creatinine (rho = 0.486; *p* = 0.012). Within diabetes strata, however, the eGFR correlations were not statistically significant (without diabetes: rho = −0.433, *p* = 0.082; with diabetes: rho = −0.083, *p* = 0.831).

Seven of 33 disease-restricted participants lacked argininosuccinic acid measurements: 4 of 21 without diabetes (19.0%) and 3 of 12 with diabetes (25.0%). Together, the secondary analyses support an exploratory association between diabetes and higher argininosuccinic acid within the disease-restricted cohort while highlighting cohort dependence, renal confounding, missingness, and the absence of statistically significant CAD effect modification.

## 3. Discussion

This exploratory targeted LC–MS/MS study identified higher estimated circulating argininosuccinic acid in patients with diabetes within a small cohort with recorded coronary vessel disease. Argininosuccinic acid was the only metabolite that remained significant after FDR correction in the disease-restricted screen, but the result was not reproduced as an FDR-significant finding in the full metabolic cohort, and formal diabetes-by-CAD interaction testing was not statistically significant. The principal finding should therefore be interpreted as a cohort-specific association requiring validation rather than as evidence of a CAD-specific metabolic phenotype or direct endothelial pathway remodeling. All argininosuccinic acid estimates fell below the lowest calibrator, limiting any inference about absolute concentrations.

### 3.1. Principal Findings

The point estimates remained positive within the analyzed sample, but this pattern does not justify describing the relationship as independent of renal function or insensitive to influential observations.

The disease-restricted screen and the full-cohort screen yielded different FDR-significant metabolites, and the formal diabetes-by-CAD interaction was nonsignificant. Accordingly, the present data do not demonstrate that argininosuccinic acid is specific to the coexistence of diabetes and CAD. The clinically restricted analysis remains useful as an exploratory comparison within patients with recorded coronary disease, but subgroup selection and small sample size must be considered when interpreting the multiplicity-adjusted result.

The seven unavailable argininosuccinic acid measurements reduced the disease-restricted analysis from 33 to 26 participants. The reasons for these seven missing values are unknown in the available analytical dataset, so selection related to concentration or sample quality cannot be excluded. The missing proportions were 4/21 (19.0%) without diabetes and 3/12 (25.0%) with diabetes.

### 3.2. Renal Function and Alternative Explanations

Renal function is a major alternative explanation because argininosuccinic acid participates in systemic amino-acid metabolism and the kidney contributes importantly to arginine and citrulline handling [26,27,28,29,30]. In the pooled disease-restricted sample, argininosuccinic acid correlated inversely with recalculated eGFR and positively with creatinine. These associations were weaker and statistically nonsignificant after stratification by diabetes status, suggesting that part of the pooled relationship may reflect between-group structure; however, the small strata sizes preclude firm conclusions.

Continuous eGFR was recalculated as a continuous value from serum creatinine, age, and sex using the 2021 race-free CKD-EPI creatinine equation rather than treating laboratory-reported values of “≥60” as precisely 60 mL/min/1.73 m^2^ [31].The diabetes coefficient remained positive after adjustment for eGFR or creatinine, but the corresponding HC3-robust confidence intervals included zero. Residual renal confounding cannot be excluded, and renal independence should not be inferred from these data.

Other plausible determinants of circulating argininosuccinic acid include hepatic urea-cycle metabolism, diet, medication exposure, systemic inflammation, and tissue-specific synthesis, degradation, transport, or utilization. The present cross-sectional serum measurements cannot separate these mechanisms.

### 3.3. Biological Context and Relation to Previous Studies

Argininosuccinic acid lies between citrulline and arginine in the ASS1/ASL recycling pathway, which is linked to endothelial NO biology [13,14,15,16,17,18,19,32,33,34,35]. Experimental and clinical studies also implicate oxidative stress, insulin signaling, arginase-related pathways, and broader disturbances of endothelial arginine/NO homeostasis in diabetic vascular dysfunction [20,21,22,23,24,25,36,37,38,39,40,41]. These observations provide a biologically plausible context for the present association, but they do not identify the source of circulating argininosuccinic acid or show that ASS1, ASL, arginase, eNOS, or NO production was altered in the study participants.

Previous metabolomic studies comparing diabetes and CAD have reported heterogeneous amino acid, lipid, and intermediary metabolic signatures, while broader diabetes and cardiovascular metabolomics studies similarly emphasize context-dependent metabolic remodeling [3,4,5,6,7,8,9,10,11,12,33,42,43,44,45,46,47,48,49,50,51,52,53,54]. The current finding should be viewed in light of that heterogeneity. It does not contradict established branched-chain or aromatic amino-acid associations, nor does it establish argininosuccinic acid as a universal biomarker. Instead, it identifies a candidate signal that may warrant targeted mechanistic and prospective study.

A future validation study should jointly measure argininosuccinic acid, citrulline, arginine, ornithine, urea, dimethylarginines, nitrate/nitrite, renal markers, and relevant medications, with standardized preanalytical procedures and, where feasible, enzyme-level or stable-isotope flux measurements. Such data would be required before assigning the circulating association to endothelial arginine recycling or NO biology.

### 3.4. Strengths, Limitations, and Implications

Strengths include targeted LC–MS/MS measurement with isotopically labelled internal standards, effect-size estimation, exact nonparametric testing, FDR correction within the disease-restricted metabolite screen, HC3-robust regression, continuous recalculation of eGFR, diabetes-stratified renal analyses, formal diabetes-by-CAD interaction testing, and influence sensitivity analyses.

The limitations are substantial. The study is retrospective and cross-sectional; the disease-restricted cohort included only 33 patients and argininosuccinic acid was available in 26; BMI and several clinical variables were incompletely recorded; several adjusted analyses were underpowered; and the top two argininosuccinic acid concentrations materially influenced precision. The vessel-count definition does not capture lesion complexity, plaque phenotype, or ischemic burden and is less granular than contemporary characterizations of chronic coronary syndrome [55]. Owing to the retrospective study design, some clinical information was incompletely documented, particularly the duration of diabetes, detailed medication exposure, and individual fasting status. Eligibility, angiographic classification, diabetes status, and preanalytical sample handling were verified against the available source clinical and laboratory records. Finally, no endothelial functional measurements, NO metabolites, pathway-enzyme measurements, flux studies, or independent validation cohort were available. Every participant-level argininosuccinic acid estimate fell below the lowest calibrator; analytical accuracy in this extrapolated range was not established, so the results are semiquantitative. The reasons for the seven missing values were unknown in the available analytical dataset, and individual fasting status was not reliably documented.

**Figure 5:**
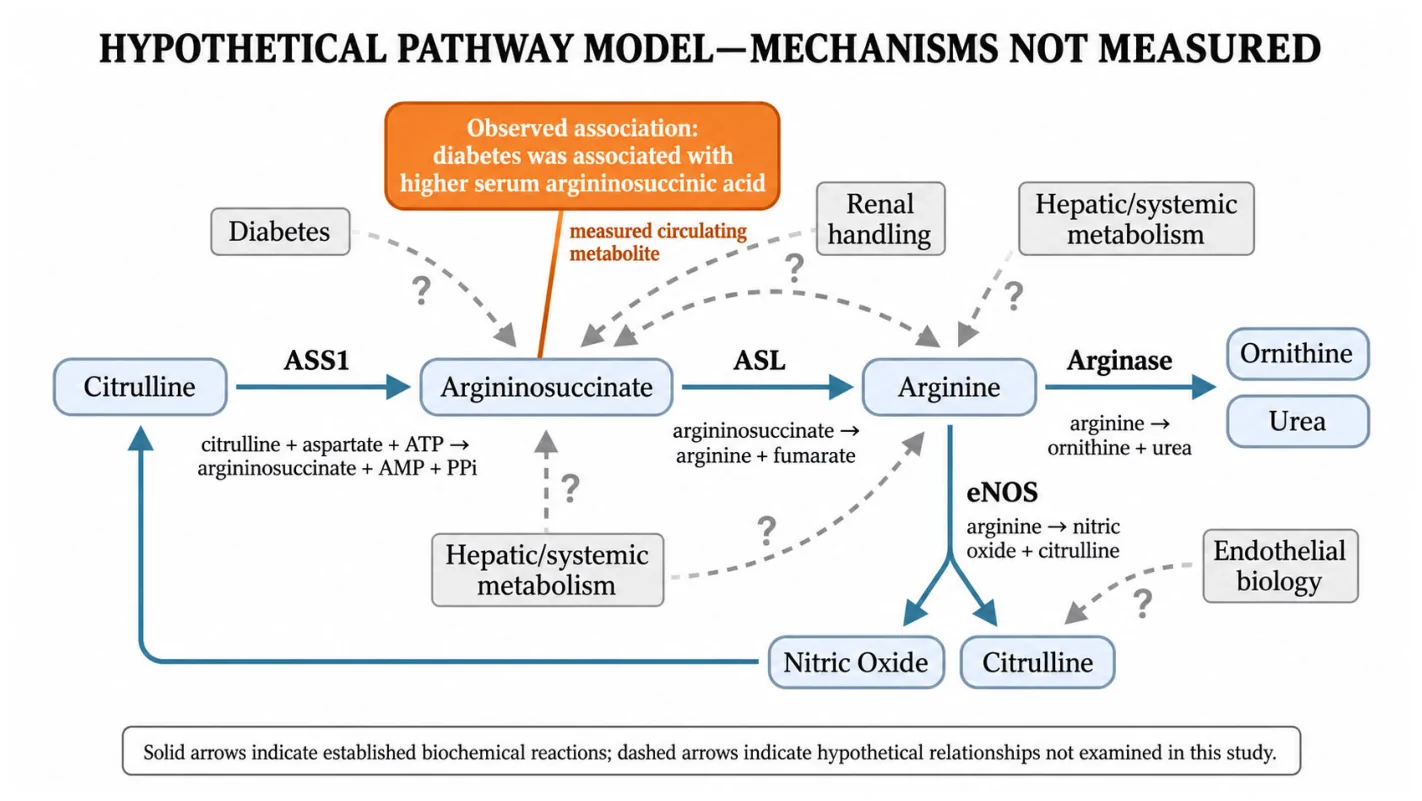
Hypothetical pathway model illustrating possible biological explanations for higher circulating argininosuccinic acid. Solid arrows indicate established biochemical reactions; dashed arrows indicate hypothetical relationships that were not examined in this study. The depicted pathway changes were not directly measured and should not be interpreted as demonstrated mechanisms.

The present result is therefore best regarded as hypothesis-generating (Figure 5). Replication in larger prospectively characterized cohorts, with detailed renal and clinical phenotyping and fully documented analytical performance, is necessary before considering mechanistic, diagnostic, or prognostic applications.

## 4. Materials and Methods

### 4.1. Study Design and Participants

This retrospective observational analysis, conducted in 2025, used the integrated clinical and amino acid database of Poznan University of Medical Sciences in Poznan, Poland. The source spreadsheet contained 111 records, including repeat records; consolidation by participant yielded 97 unique patients. Argininosuccinic acid estimates were available for 73 of these patients. Coronary vessel count was recorded for 70 patients: 37 had a count of 0, and 33 had at least one diseased vessel (21 without diabetes and 12 with diabetes). Argininosuccinic acid was available for 26 of the 33 disease-restricted patients (17 without diabetes and 9 with diabetes). The conventional multivessel-disease sensitivity group comprised 18 patients with vessel count ≥ 2. The restricted comparison was exploratory and does not establish CAD specificity.

Eligibility criteria. Adult patients were eligible for inclusion if they were referred for evaluation of chronic coronary syndrome and underwent coronary angiography at the study center during the predefined recruitment period in 2025. Eligibility required the availability of clinical data sufficient to determine diabetes status and coronary angiographic findings, together with a serum sample suitable for targeted amino acid and related metabolite analysis. For the primary disease-restricted analysis, patients were additionally required to have angiographically documented coronary artery disease, defined as at least one coronary vessel with significant stenosis (>70%, or ≥50% for the left main coronary artery). Patients were classified according to the presence or absence of established type 2 diabetes mellitus. Diabetes status was based on the clinical diagnosis recorded in the source medical documentation and/or ongoing glucose-lowering treatment, as applicable to the original clinical records.

Exclusion criteria. Patients were excluded if they presented with an acute coronary syndrome; had undergone previous percutaneous coronary intervention or coronary artery bypass grafting; had type 1 diabetes mellitus, secondary diabetes, or newly diagnosed type 2 diabetes mellitus; followed a restrictive diet or had documented food allergies considered potentially relevant to metabolic profiling; or lacked sufficient clinical or angiographic information to determine eligibility. Records representing repeat observations of the same participant were consolidated at the participant level rather than treated as independent observations. Participants without an available measurement for a specific metabolite were excluded only from analyses requiring that metabolite and were not removed from the overall clinical cohort.

### 4.2. Clinical and Angiographic Data

Demographic, anthropometric, laboratory, comorbidity, medication, echocardiographic, and angiographic variables were obtained from the integrated database and available records. Diabetes status was defined by the recorded binary DIABETES variable (0, no diabetes; 1, diabetes). The analysis database included age, sex, BMI, blood-cell counts, hemoglobin, creatinine, laboratory-reported eGFR, glucose, HbA1c, lipid variables, uric acid, major comorbidities, smoking status when available, and recorded medication classes including beta-blockers, statins, ACE inhibitors, ARBs, calcium-channel blockers, mineralocorticoid receptor antagonists, diuretics, and SGLT2 inhibitors. The available clinical description classified diabetes cases as established type 2 diabetes.

Diabetes status was determined retrospectively from the source clinical records. Type 2 diabetes mellitus (T2DM) was considered established when a diagnosis of T2DM was documented in the patient’s medical history before the index hospitalization and/or the patient was receiving established glucose-lowering therapy for previously diagnosed T2DM. Patients with type 1 diabetes, secondary diabetes, or diabetes newly diagnosed during the index hospitalization were excluded. The exact date of the initial T2DM diagnosis was not consistently available in the retrospective records; therefore, diabetes duration could not be reliably determined and was not included in the analyses.

Continuous eGFR was recalculated using the 2021 race-free CKD-EPI creatinine equation: eGFR = 142 × min(SCr/κ, 1)^α^ × max(SCr/κ, 1)^−1.200^ × 0.9938^Age^ × 1.012 (if female), with κ = 0.7 and α = −0.241 for women and κ = 0.9 and α = −0.302 for men [31]. Laboratory-reported values of “≥60” were therefore not treated as exactly 60 mL/min/1.73 m^2^, and the recalculated continuous eGFR was used in revised renal analyses. Blood-cell measurements were obtained using a Sysmex hematology analyzer (Sysmex Europe, Norderstedt, Germany). All measurements were performed in the reference laboratory of the central hospital. Coronary disease burden was represented by the recorded angiographic vessel-disease count (0–3); count ≥ 1 defined the disease-restricted cohort, and count ≥ 2 defined conventional multivessel disease. Stenoses greater than 70% were categorized as significant (except for left main stenosis of at least 50%). Normal coronary arteries were defined as those with lumen narrowing of less than 20%. There were no patients with coronary stenosis between 20–70% in the analyzed group. The coronary angiography was performed by a team of experienced hemodynamists at a reference center.

### 4.3. Amino-Acid and Related-Metabolite Measurement

#### 4.3.1. Chemicals and Reagents

All reagents used for amino-acid analysis were supplied by Chromsystems Instruments & Chemicals GmbH (Gräfelfing, Germany). Quantification was performed with the MassChrom Amino Acid Analysis in Plasma/Serum kit for LC–MS/MS (order no. 75111), which provided mobile phase A (75001), mobile phase B (75002), rinsing solution (75009), the protein-precipitation reagent (75105), the stable-isotope-labeled internal standard set (75146), and the 1.5 mL reaction vials in which sample preparation was performed (33006). Every analyte in the panel was assigned its own stable-isotope-labeled analogue. Chromatographic separation was performed on the dedicated MassChrom analytical column for amino-acid analysis (75100). Calibration was based on the 3PLUS1 Multilevel Plasma Calibrator Set for Amino Acid Analysis (75128), and assay performance was monitored with lyophilized human-plasma-based MassCheck Amino Acid Analysis Plasma Controls at three concentration levels (Level I, 0471; Level II, 0472; Level III, 0473). The mass spectrometer was tuned using Chromsystems tuning mixes 1–5 (75015–75019).

#### 4.3.2. Specimen Handling and Sample Preparation

Serum was used as the analytical matrix. Following blood collection, samples were processed according to the standardized institutional laboratory protocol. Serum was separated from the cellular fraction within 15 min of collection, aliquoted, and stored at −80 °C until analysis. Samples were thawed immediately before LC–MS/MS analysis and were not subjected to repeated freeze–thaw cycles before metabolite measurement. After thawing, samples were prepared according to the commercial kit protocol. Serum (25 µL) was combined with 50 µL of reconstituted isotopically labeled internal-standard solution and 400 µL of the supplied precipitation reagent in a 1.5 mL reaction vial. The mixture was vortexed for 30 s and centrifuged at 16,000× *g* for 5 min, after which 200 µL of supernatant was transferred to an autosampler vial. Calibrators and controls were processed and analyzed in the same manner as study samples. All measurements were performed in the reference laboratory of the central hospital.

#### 4.3.3. Liquid Chromatography–Tandem Mass Spectrometry

Measurements were performed using a triple-quadrupole/linear ion-trap mass spectrometer (SCIEX, Framingham, MA, USA) coupled to an Agilent 1260 Infinity II liquid chromatograph (Agilent Technologies, Santa Clara, CA, USA). Separation used the dedicated MassChrom amino-acid analytical column at 25 °C with a 5 µL injection volume.

Chromatographic separation was performed using the dedicated MassChrom analytical column for amino-acid analysis (Chromsystems Instruments & Chemicals GmbH, Gräfelfing, Germany; order no. 75100), maintained at 25 °C. The column was supplied as a proprietary component of the validated commercial analytical system; column dimensions and particle size were not specified in the laboratory method documentation available for this retrospective analysis.

The assay used positive-ion electrospray ionization and multiple-reaction monitoring, with a total run time of 20.5 min. The chromatographic solvent and flow-rate program is provided in Supplementary Table S10, and scheduled MRM transitions, retention times, compound-dependent parameters, and source parameters are provided in Supplementary Table S11.

#### 4.3.4. Calibration, Quantification, and Quality Control

Peak integration and concentration calculation were performed in SCIEX OS 3.3.0.12027 using a targeted quantitation method in which every analyte was referenced to its own stable-isotope-labeled internal standard. Concentrations were derived from analyte-to-internal-standard peak-area ratios by linear regression with 1/x weighting against the three levels of the 3PLUS1 multilevel plasma calibrator set and are reported as µmol/L. The available quality-control summary lists calibrator lot 3524, MassCheck control lot 3823, and internal-standard mix lots 4223 and 4124.

The available analytical summary describes randomized sample coding and acquisition, three calibrator levels, three MassCheck control levels, and six Level II control determinations. Across 45 analytes listed in Table S9, the six reported Level II coefficients of variation ranged from 0.6% to 9.3% (median, 3.0%). The summary indicates no drift correction and no quantifiable carryover in blanks after the highest calibrator.

Study samples were analyzed using coded identifiers and in randomized acquisition order. The analytical sequence included study samples, three calibration levels, three MassCheck quality-control levels, and six Level II quality-control determinations distributed throughout the sequence to assess within-batch precision. Clinical group status was not used to determine the analytical sequence or during LC–MS/MS data acquisition and quantitative processing. Formal blinding of laboratory personnel to all clinical information was not documented in the available retrospective laboratory records.

Calibration used the three levels of the 3PLUS1 plasma calibrator set, and the analyte-specific calibration range, given by the nominal lot-specific concentrations of the lowest (Cal1) and highest (Cal3) calibrator, is reported for every analyte in Table S9. Run acceptance was assessed against the conventional bioanalytical criteria of back-calculated calibrator concentrations within 15% of nominal, or 20% at the lowest calibration level, and control recoveries within 15% of nominal. All calibrators and all control determinations across the 45 reported analytes met these limits, with calibrator accuracies of 89.6% to 109.2% and control recoveries of 86.2% to 111.8%, which documents the accuracy of the assay across the calibrated range. Linearity was assessed from the fitted calibration curves, which relate the analyte-to-internal-standard peak-area ratio to concentration by linear regression with 1/x weighting. Coefficients of determination ranged from 0.9939 to 1.0000 across the 45 analytes, and the curve equation for each analyte is given in Table S9. Because the calibration comprised only three levels, these coefficients are reported alongside, rather than in place of, the back-calculated calibrator accuracies. Within-batch precision, taken as the coefficient of variation in the six Level II control determinations distributed across the sequence, ranged from 0.6% to 9.3% (median, 3.0%). Inter-assay precision is not documented in the available single-batch quality-control summary. Because the manufacturer does not declare limits of detection, analyte-specific limits of detection and quantification were estimated from the signal-to-noise ratio of the lowest calibrator, using concentrations corresponding to signal-to-noise ratios of 3 and 10, respectively. These extrapolated estimates are listed in Table S9.

All 73 participant-level argininosuccinic acid estimates included in the full-cohort analysis (0.116–1.011 µmol/L) were below the lowest calibrator (2.64 µmol/L); these are extrapolated, semiquantitative estimates rather than validated absolute concentrations. Each estimate exceeded the signal-to-noise-derived estimated LOQ (0.07368 µmol/L), but that estimate does not establish accuracy or precision below the calibrated range. The reported fitted line, y = 0.1323x − 0.00995 (r^2^ = 0.99951), crosses zero response near 0.075 µmol/L. At the median participant-level estimate of 0.174 µmol/L, the absolute intercept is about 43% of the predicted zero-intercept response, rising to about 65% at the lowest estimate. These calculations further caution against absolute quantification or biomarker claims.

### 4.4. Study Outcomes and Analysis Populations

The objective was to explore diabetes-associated differences in amino acids and related metabolites within patients with recorded coronary vessel disease. The main disease-restricted endpoint was the between-group difference for each metabolite among patients with vessel count ≥ 1. Argininosuccinic acid emerged as the leading FDR-significant signal in this screen and was subsequently evaluated using adjusted and sensitivity analyses. Secondary/contextual analyses included the full metabolic cohort, the conventional multivessel-disease subgroup, cohort accounting and missingness, renal-function associations, observation-level influence, and formal diabetes-by-CAD interaction testing. These analyses were interpreted as exploratory and were not used to infer CAD-specificity.

### 4.5. Statistical Analysis

Continuous variables were summarized as medians and interquartile ranges. For metabolite screening, two-sided exact Wilcoxon rank-sum tests were implemented with coin::wilcox_test using the conditional exact distribution [56]; this implementation accommodates tied observations through the conditional permutation distribution. Cliff’s delta was calculated with the effsize package, with positive values indicating higher concentrations in diabetes [57]. Of the 45 analyte columns in the participant-level database, 39 permitted valid two-group tests in the disease-restricted cohort; these 39 *p*-values were adjusted using the Benjamini–Hochberg false-discovery-rate procedure [58]. In the full metabolic cohort, 41 analytes permitted valid two-group tests and were adjusted in a separate 41-test family. The acquisition method included three additional analytes (leucine, isoleucine, and phosphoserine) for which no participant-level concentration columns were present; they were not screened. The full-cohort screen and other subgroup/sensitivity analyses were treated as contextual exploratory analyses rather than as part of a single confirmatory multiplicity family.

For argininosuccinic acid, the metabolite was standardized once to a z-score using all 26 available observations in the disease-restricted cohort; this same standardized outcome was retained across model-specific complete-case subsets. HC3 heteroscedasticity-consistent standard errors were used for the revised regression inference [59]. Models included crude, age and sex, age/sex/BMI, recalculated eGFR, age/sex/creatinine, age/sex/BMI/creatinine, and a log-transformed outcome that was subsequently standardized. Sensitivity analyses excluded the two highest argininosuccinic acid concentrations and restricted the analysis to recalculated eGFR ≥ 60 mL/min/1.73 m^2^. Leave-one-out influence analyses were also performed.

Spearman rank correlations evaluated argininosuccinic acid with recalculated eGFR and creatinine both in the pooled disease-restricted sample and within diabetes strata. A formal diabetes-by-CAD interaction was assessed in participants with available argininosuccinic acid and angiographic disease status using standardized argininosuccinic acid as the outcome, with crude, age- and sex-adjusted, and log-outcome models. Missing data were not imputed. Analyses were performed in R version 4.6.0 using readxl, dplyr, tidyr, purrr, coin, effsize, broom, lmtest, sandwich, ggplot2, openxlsx, officer, and flextable.

## 5. Conclusions

In this small exploratory cohort with recorded coronary vessel disease, diabetes was associated with higher estimated circulating argininosuccinic acid, which was the only metabolite to remain significant after FDR correction in the disease-restricted screen. The association remained directionally positive across multiple sensitivity analyses, but renal adjustment, influential observations, missingness, and limited sample size reduced precision, and formal interaction testing did not establish CAD specificity. All estimates were extrapolated below the lowest calibrator and are semiquantitative; the finding should therefore be considered hypothesis-generating. Larger prospectively characterized cohorts with detailed renal phenotyping, complete clinical covariates, rigorous analytical validation, and direct pathway measurements are required before mechanistic or biomarker interpretations can be supported.

## Figures and Tables

**Table 1:** Clinical characteristics, comorbidities, and available medications according to diabetes status in the disease-restricted cohort.

Characteristic	Without Diabetes	With Diabetes	Exact *p*-Value
Age, years	64.00 [59.00–73.00] (*n* = 21)	70.00 [63.50–73.50] (*n* = 12)	0.511
BMI, kg/m^2^	25.89 [24.90–28.87] (*n* = 15)	27.16 [26.16–29.54] (*n* = 10)	0.255
White blood cell count, 10^9^/L	6.50 [5.44–7.54] (*n* = 21)	9.21 [8.29–10.25] (*n* = 12)	0.007
Neutrophils, 10^9^/L	3.90 [2.99–5.04] (*n* = 21)	6.06 [5.03–6.51] (*n* = 12)	0.015
Lymphocytes, 10^9^/L	1.66 [1.34–2.21] (*n* = 21)	2.31 [1.97–2.63] (*n* = 12)	0.052
Monocytes, 10^9^/L	0.51 [0.39–0.72] (*n* = 21)	0.77 [0.55–0.88] (*n* = 12)	0.045
Hemoglobin, mmol/L	9.00 [8.50–9.40] (*n* = 21)	9.45 [8.30–9.65] (*n* = 12)	0.499
Creatinine, mg/dL	0.84 [0.73–0.96] (*n* = 21)	1.00 [0.77–1.33] (*n* = 12)	0.270
eGFR (2021 CKD-EPI), mL/min/1.73 m^2^	91.80 [77.20–97.50] (*n* = 21)	82.05 [49.70–103.25] (*n* = 12)	0.313
Serum glucose, mg/dL	97.00 [89.00–102.00] (*n* = 21)	122.50 [105.00–134.25] (*n* = 12)	0.010
HbA1c, %	5.90 [5.50–6.10] (*n* = 7)	7.00 [6.53–7.43] (*n* = 8)	0.010
Total cholesterol, mg/dL	157.00 [128.00–212.00] (*n* = 21)	145.00 [121.00–179.00] (*n* = 10)	0.582
LDL cholesterol, mg/dL	66.00 [57.00–109.00] (*n* = 21)	82.50 [50.00–97.50] (*n* = 12)	0.832
HDL cholesterol, mg/dL	56.00 [47.00–64.00] (*n* = 21)	42.00 [37.25–46.75] (*n* = 12)	0.026
Triglycerides, mg/dL	79.00 [61.00–127.00] (*n* = 21)	105.50 [98.75–161.75] (*n* = 12)	0.020
Uric acid, mg/dL	4.90 [4.30–6.30] (*n* = 21)	6.50 [5.38–6.75] (*n* = 12)	0.184
Male sex	13/21 (61.9%)	7/12 (58.3%)	1.000
Hypertension	17/21 (81.0%)	11/12 (91.7%)	0.630
Dyslipidemia	20/21 (95.2%)	10/12 (83.3%)	0.538
Kidney disease	2/21 (9.5%)	3/12 (25.0%)	0.328
COPD	0/21 (0.0%)	2/12 (16.7%)	0.125
Peripheral artery disease	0/21 (0.0%)	1/12 (8.3%)	0.364
Ischemic heart disease	21/21 (100.0%)	12/12 (100.0%)	1.000
Atrial fibrillation	2/21 (9.5%)	1/12 (8.3%)	1.000
Heart failure	7/21 (33.3%)	4/12 (33.3%)	1.000
Prior myocardial infarction	2/21 (9.5%)	2/12 (16.7%)	0.610
Beta-blocker	13/21 (61.9%)	10/12 (83.3%)	0.259
Statin	17/21 (81.0%)	9/12 (75.0%)	0.686
ACE inhibitor	10/21 (47.6%)	5/12 (41.7%)	1.000
ARB	6/21 (28.6%)	3/12 (25.0%)	1.000
Calcium-channel blocker	7/21 (33.3%)	3/12 (25.0%)	0.710
Mineralocorticoid receptor antagonist	6/21 (28.6%)	4/12 (33.3%)	1.000
Diuretic	10/21 (47.6%)	4/12 (33.3%)	0.486
SGLT2 inhibitor	5/21 (23.8%)	6/12 (50.0%)	0.149
Smoking status available	2/21 (9.5%)	3/12 (25.0%)	—
Former smoker among recorded	1/2 (50.0%)	2/3 (66.7%)	—
Never smoker among recorded	1/2 (50.0%)	1/3 (33.3%)	—

Abbreviations and units: ACE, angiotensin-converting enzyme; ARB, angiotensin II receptor blocker; BMI, body mass index; CKD-EPI, Chronic Kidney Disease Epidemiology Collaboration; COPD, chronic obstructive pulmonary disease; eGFR, estimated glomerular filtration rate; HbA1c, glycated hemoglobin A1c; HDL, high-density lipoprotein; LDL, low-density lipoprotein; SGLT2, sodium–glucose cotransporter 2; *n*, number of observations; kg/m^2^, kilograms per square meter; 10^9^/L, 10^9^ cells per liter; mmol/L, millimoles per liter; mg/dL, milligrams per deciliter; mL/min/1.73 m^2^, milliliters per minute per 1.73 square meters; %, percent. Data are presented as median [interquartile range] or n/N (%), as appropriate.

**Table 2:** Leading amino acid and related metabolite differences according to diabetes status in the disease-restricted cohort.

Amino Acid/Metabolite	Without Diabetes, µmol/L	With Diabetes, µmol/L	Cliff’s Delta	Exact *p*-Value	FDR q
Argininosuccinic acid	0.15 [0.13–0.19] (*n* = 17)	0.28 [0.21–0.34] (*n* = 9)	0.765	<0.001	0.035
α-Aminoadipic acid	1.03 [0.89–1.31] (*n* = 20)	1.57 [1.12–2.45] (*n* = 12)	0.458	0.032	0.355
Alloisoleucine	1.77 [1.29–2.11] (*n* = 21)	2.23 [1.73–2.51] (*n* = 12)	0.452	0.033	0.355
Alanine	399.85 [306.70–472.77] (*n* = 21)	491.84 [415.31–533.80] (*n* = 12)	0.444	0.036	0.355
Lysine	194.21 [170.83–204.26] (*n* = 21)	198.42 [187.47–221.25] (*n* = 12)	0.389	0.069	0.539
Homocitrulline	0.73 [0.66–0.85] (*n* = 21)	0.83 [0.75–1.08] (*n* = 12)	0.353	0.098	0.638
Proline	199.81 [165.42–224.22] (*n* = 21)	258.24 [198.20–271.19] (*n* = 12)	0.333	0.122	0.639
4-Hydroxyproline	12.39 [9.10–16.70] (*n* = 21)	15.90 [12.16–23.52] (*n* = 12)	0.325	0.131	0.639
Tyrosine	65.68 [60.83–69.06] (*n* = 21)	57.18 [51.30–66.28] (*n* = 12)	−0.310	0.152	0.657
Hydroxylysine	0.04 [0.01–0.07] (*n* = 5)	0.20 [0.11–0.31] (*n* = 4)	0.600	0.175	0.663

Abbreviations: FDR, false discovery rate; *n*, number of observations; *p*, probability value; q, FDR-adjusted *p*-value. Cliff’s delta (δ), a non-parametric effect-size measure quantifying the degree of separation between two groups; positive values indicate higher concentrations in the diabetes group, and negative values indicate lower concentrations in the diabetes group.

**Table 3:** HC3-robust adjusted and sensitivity analyses for argininosuccinic acid.

Model	*n*	Estimate (SD Units)	95% CI	*p*-Value	SE Method
Crude	26	1.199	0.185 to 2.213	0.022	HC3 robust
Age + sex	26	1.182	0.098 to 2.265	0.034	HC3 robust
Age + sex + BMI	19	1.285	−0.151 to 2.721	0.076	HC3 robust
DM + recalculated eGFR	26	1.159	−0.027 to 2.346	0.055	HC3 robust
Age + sex + creatinine	26	1.180	−0.094 to 2.455	0.068	HC3 robust
Age + sex + BMI + creatinine	19	1.196	−0.975 to 3.367	0.255	HC3 robust
Log outcome: age + sex	26	1.246	0.292 to 2.201	0.013	HC3 robust
Exclude 2 highest ASA values: age + sex	24	0.532	−0.022 to 1.086	0.059	HC3 robust
Recalculated eGFR ≥ 60: age + sex	20	1.149	−0.750 to 3.047	0.218	HC3 robust

Abbreviations: ASA, argininosuccinic acid; BMI, body mass index; CI, confidence interval; DM, diabetes mellitus; eGFR, estimated glomerular filtration rate; HC3, heteroskedasticity-consistent covariance matrix estimator type 3; *n*, number of observations; SD, standard deviation; SE, standard error. Estimates are expressed in SD units. HC3 robust standard errors were used to account for potential heteroskedasticity.

## Data Availability

Data supporting the presented results will be available upon reasonable request from the corresponding author.

## Supplementary Materials

The following supporting information can be downloaded at: https://www.mdpi.com/article/10.3390/ijms27198766/s1.

## Abbreviations

The following abbreviations are used in this manuscript:

ASL	Argininosuccinate Lyase
ASS1	Argininosuccinate Synthase 1
BMI	Body Mass Index
CAD	Coronary Artery Disease
CI	Confidence Interval
CKD	Chronic Kidney Disease
eGFR	Estimated Glomerular Filtration Rate
eNOS	Endothelial Nitric Oxide Synthase
FDR	False Discovery Rate
HbA1c	Glycated Hemoglobin
HC3	Heteroscedasticity-Consistent Standard Error Estimator (HC3)
HDL	High-Density Lipoprotein
IQR	Interquartile Range
LDL	Low-Density Lipoprotein
NMR	Nuclear Magnetic Resonance
NO	Nitric Oxide
Q1	First Quartile
Q3	Third Quartile
SD	Standard Deviation
T2DM	Type 2 Diabetes Mellitus
CKD-EPI	Chronic Kidney Disease Epidemiology Collaboration
LC–MS/MS	Liquid Chromatography–Tandem Mass Spectrometry
