# Supplementary material for: Serum Argininosuccinic Acid Is Associated with Diabetes in Patients with Coronary Artery Disease: An Exploratory Targeted LC–MS/MS Study
Source: Int J Mol Sci. 2026 Sep 30;27(19):8766. doi: 10.3390/ijms27198766 (PMC13664958; doi:10.3390/ijms27198766)
Supplement: Supplementary file 1 [file ijms-27-08766-s001.zip › ijms-4521652-supplementary.pdf]

Supplementary Materials

Serum Argininosuccinic Acid Is Associated with Diabetes in Patients with Coronary Artery Disease: An Exploratory Targeted LC–MS/MS Study

Beata Krasińska, Minseung Kang, Siwon Baek, et al.

Table S1. Full metabolic cohort clinical characteristics according to diabetes status

Characteristic	Unit	Without diabetes	n	With diabetes	n	Exact p-value
Age	years	67.00 [56.75–73.00]	68	70.00 [63.00–75.00]	29	0.152
BMI	kg/m²	26.03 [24.38–29.40]	45	29.71 [26.89–32.16]	23	0.004
White blood cell count	10^9/L	6.35 [5.22–7.87]	68	8.06 [6.60–9.59]	29	0.004
Neutrophils	10^9/L	4.05 [2.91–5.24]	68	5.04 [3.68–6.18]	29	0.015
Lymphocytes	10^9/L	1.59 [1.30–2.01]	68	1.98 [1.55–2.41]	29	0.017
Monocytes	10^9/L	0.55 [0.41–0.72]	68	0.63 [0.51–0.73]	29	0.119
Hemoglobin	mmol/L	8.90 [8.40–9.33]	68	8.90 [8.00–9.50]	29	0.648
Creatinine	mg/dL	0.87 [0.70–1.00]	67	0.94 [0.76–1.15]	29	0.160
eGFR (2021 CKD-EPI)	mL/min/1.73 m²	92.20 [75.75–99.35]	67	85.30 [55.50–94.90]	29	0.044
Serum glucose	mg/dL	96.00 [87.75–102.50]	68	116.00 [102.00–135.00]	29	<0.001
HbA1c	%	5.60 [5.45–5.95]	11	6.70 [6.10–7.40]	21	<0.001
Total cholesterol	mg/dL	160.00 [131.25–211.25]	66	150.00 [123.00–174.00]	27	0.078
LDL cholesterol	mg/dL	76.50 [61.25–122.75]	66	74.00 [50.00–96.00]	29	0.215
HDL cholesterol	mg/dL	53.50 [46.25–65.00]	66	42.00 [38.00–47.00]	29	<0.001
Triglycerides	mg/dL	84.00 [68.00–119.00]	66	114.00 [98.00–152.00]	29	<0.001
Uric acid	mg/dL	5.30 [4.30–6.20]	66	5.60 [4.50–6.70]	29	0.217

Data are median [interquartile range]. Exact p-values were obtained from two-sided exact Wilcoxon rank-sum tests. eGFR is the continuous 2021 race-free CKD-EPI creatinine estimate recalculated from serum creatinine, age, and sex.

Abbreviations: BMI, body mass index; CKD-EPI, Chronic Kidney Disease Epidemiology Collaboration; eGFR, estimated glomerular filtration rate; HbA1c, glycated hemoglobin; HDL, high-density lipoprotein; IQR, interquartile range; LDL, low-density lipoprotein.

Table S2. Complete amino-acid and related-metabolite comparison in the full metabolic cohort.

Metabolite	n no DM	No diabetes, median [IQR] (μmol/L)	n DM	Diabetes, median [IQR] (μmol/L)	Cliff's δ	Exact p	FDR q
Ethanolamine	68	10.71 [8.97–12.04]	29	8.70 [7.61–10.57]	-0.412	0.001	0.048
Argininosuccinic Acid	50	0.16 [0.14–0.20]	23	0.21 [0.16–0.31]	0.388	0.008	0.129
Homocitrulline	68	0.78 [0.68–0.87]	29	0.89 [0.75–1.22]	0.323	0.012	0.129
Alloisoleucine	68	1.82 [1.37–2.16]	29	2.17 [1.72–2.50]	0.319	0.013	0.129
Arginine	68	89.02 [78.22–98.59]	29	81.16 [71.26–90.21]	-0.273	0.034	0.196
Alpha Aminoadipic Acid	67	0.91 [0.82–1.32]	29	1.20 [0.83–1.75]	0.270	0.036	0.196
Serine	68	147.46 [130.70–155.60]	29	128.57 [112.43–153.71]	-0.262	0.042	0.196
3 Methylhistidine	68	9.02 [6.38–14.82]	29	11.43 [7.91–22.67]	0.261	0.043	0.196
1-Methylhistidine	68	5.36 [4.54–6.72]	29	6.63 [4.74–8.55]	0.254	0.049	0.196
Threonine	68	118.41 [106.75–132.99]	29	110.54 [94.42–124.43]	-0.253	0.050	0.196
Glutamine	68	653.34 [598.14–697.40]	29	618.96 [559.57–669.24]	-0.249	0.053	0.196
Alanine	68	402.32 [354.30–475.62]	29	429.81 [384.35–530.08]	0.242	0.060	0.203
Citrulline	68	31.87 [28.50–38.36]	29	29.71 [23.09–34.67]	-0.238	0.064	0.203
Asparagine	68	45.84 [41.66–51.11]	29	42.97 [36.86–49.49]	-0.232	0.072	0.211
Taurine	68	158.13 [132.93–188.35]	29	144.11 [116.58–172.49]	-0.220	0.088	0.241
Valine	68	237.13 [215.44–258.88]	29	249.61 [223.77–277.32]	0.214	0.097	0.249
Aspartic Acid	68	29.17 [23.38–35.34]	29	26.40 [18.50–28.77]	-0.210	0.104	0.251
Cystathionine	68	0.90 [0.80–0.97]	29	0.92 [0.84–1.17]	0.192	0.136	0.311
Phosphoethanolamine	64	1.78 [1.17–3.17]	26	1.47 [1.33–1.89]	-0.180	0.185	0.396
Ornithine	68	100.70 [84.93–112.85]	29	93.23 [69.21–112.29]	-0.168	0.193	0.396
Cysteine Sulfate	68	2.13 [2.00–2.33]	29	2.11 [1.97–2.19]	-0.158	0.223	0.404
Cystine	68	51.78 [38.84–63.28]	29	54.68 [43.65–65.32]	0.150	0.247	0.404
Sarcosine	68	1.44 [1.26–1.93]	29	1.40 [1.06–1.75]	-0.149	0.250	0.404
Hydroxylysine	12	0.06 [0.03–0.07]	9	0.13 [0.05–0.24]	0.306	0.254	0.404
Tyrosine	68	63.67 [55.04–69.54]	29	59.97 [51.58–67.67]	-0.144	0.267	0.404
Adenosylhomocysteine	44	0.14 [0.13–0.15]	24	0.15 [0.14–0.16]	0.164	0.270	0.404
Tryptophane	68	68.15 [60.27–76.48]	29	64.10 [57.97–75.84]	-0.142	0.273	0.404
Alpha Aminobutyric Acid	68	20.28 [16.35–24.94]	29	21.77 [17.93–25.68]	0.141	0.277	0.404

Metabolite	n no DM	No diabetes, median [IQR] (μmol/L)	n DM	Diabetes, median [IQR] (μmol/L)	Cliff's δ	Exact p	FDR q
Lysine	68	183.73 [169.02–207.83]	29	187.54 [178.84–216.72]	0.136	0.295	0.404
Methionine	68	24.34 [21.81–27.84]	29	22.87 [20.86–26.94]	-0.135	0.295	0.404
Glycine	68	240.62 [208.30–273.86]	29	226.30 [198.91–274.99]	-0.115	0.377	0.499
Beta Aminoisobutyric Acid	68	1.98 [1.29–2.48]	29	2.17 [1.53–2.52]	0.100	0.439	0.562
Proline	68	198.36 [156.45–222.44]	29	203.16 [166.80–257.23]	0.091	0.483	0.600
4-Hydroxyproline	68	12.15 [9.09–15.43]	29	13.21 [9.56–17.28]	0.078	0.548	0.660
Glutamic Acid	68	92.05 [71.73–117.66]	29	96.39 [73.32–118.63]	0.051	0.698	0.817
Homocystine	13	0.05 [0.05–0.12]	6	0.06 [0.05–0.06]	-0.115	0.717	0.817
Phenylalanine	68	71.40 [62.29–79.59]	29	70.64 [64.18–76.02]	-0.038	0.775	0.859
Histidine	68	83.50 [80.99–90.25]	29	85.09 [76.76–92.02]	0.024	0.854	0.921
Beta Alanine	68	2.42 [1.96–3.12]	29	2.57 [2.12–3.19]	0.010	0.939	0.969
Pipecolic Acid	68	1.34 [1.13–2.07]	29	1.46 [1.13–1.72]	-0.009	0.945	0.969
Saccharopine	3	0.17 [0.15–0.25]	9	0.20 [0.18–0.23]	0.037	1.000	1.000
Acetylotyrosine	1	0.12 [0.12–0.12]	0	—	—	—	—

Abbreviations: DM, diabetes mellitus; FDR, false discovery rate; IQR, interquartile range; q, false-discovery-rate-adjusted p-value.

Metabolite	n no DM	No diabetes, median [IQR] (μmol/L)	n DM	Diabetes, median [IQR] (μmol/L)	Cliff's δ	Exact p	FDR q
Carnosine	0	—	0	—	—	—	—
Gamma Aminobutyric Acid	0	—	0	—	—	—	—
Anserine	0	—	0	—	—	—	—

Positive Cliff's delta indicates higher concentrations in diabetes. FDR q values were calculated using the Benjamini-Hochberg procedure across the metabolite-wide screen. Metabolites without sufficient observations for a two-group comparison are shown with em dashes. The full-cohort FDR family comprised 41 valid tests. Argininosuccinic acid values are semiquantitative estimates extrapolated below the lowest calibrator.

Table S3. Complete amino-acid and related-metabolite comparison in the primary CAD cohort

Metabolite	n no DM	No diabetes, median [IQR] (μmol/L)	n DM	Diabetes, median [IQR] (μmol/L)	Cliff's δ	Exact p	FDR q
Argininosuccinic Acid	17	0.15 [0.13–0.19]	9	0.28 [0.21–0.34]	0.765	<0.001	0.035
Alpha Aminoadipic Acid	20	1.03 [0.89–1.31]	12	1.57 [1.12–2.45]	0.458	0.032	0.355
Alloisoleucine	21	1.77 [1.29–2.11]	12	2.23 [1.73–2.51]	0.452	0.033	0.355
Alanine	21	399.85 [306.70–472.77]	12	491.84 [415.31–533.80]	0.444	0.036	0.355
Lysine	21	194.21 [170.83–204.26]	12	198.42 [187.47–221.25]	0.389	0.069	0.539
Homocitrulline	21	0.73 [0.66–0.85]	12	0.83 [0.75–1.08]	0.353	0.098	0.638
Proline	21	199.81 [165.42–224.22]	12	258.24 [198.20–271.19]	0.333	0.122	0.639
4-Hydroxyproline	21	12.39 [9.10–16.70]	12	15.90 [12.16–23.52]	0.325	0.131	0.639
Tyrosine	21	65.68 [60.83–69.06]	12	57.18 [51.30–66.28]	-0.310	0.152	0.657
Hydroxylysine	5	0.04 [0.01–0.07]	4	0.20 [0.11–0.31]	0.600	0.175	0.663
Glycine	21	237.73 [222.09–274.53]	12	272.72 [233.27–304.12]	0.286	0.187	0.663
Adenosylhomocysteine	18	0.14 [0.13–0.15]	8	0.15 [0.14–0.16]	0.306	0.232	0.707
1-Methylhistidine	21	5.50 [4.89–6.44]	12	6.84 [4.60–9.51]	0.246	0.258	0.707
Glutamic Acid	21	95.27 [64.81–109.10]	12	105.66 [92.52–133.62]	0.246	0.258	0.707
Histidine	21	83.13 [81.05–90.95]	12	90.31 [82.99–101.10]	0.230	0.291	0.707
Valine	21	245.51 [218.89–263.89]	12	257.87 [236.09–288.44]	0.230	0.291	0.707
Cystathionine	21	0.95 [0.83–1.07]	12	0.98 [0.88–1.67]	0.222	0.308	0.707
Cysteine Sulfate	21	2.03 [1.94–2.31]	12	2.15 [2.05–2.31]	0.206	0.345	0.748
Glutamine	21	672.90 [619.94–718.99]	12	665.07 [606.55–687.90]	-0.183	0.405	0.832
Ethanolamine	21	9.88 [8.92–11.69]	12	9.93 [7.76–11.29]	-0.167	0.449	0.833
Threonine	21	118.40 [104.77–130.11]	12	109.00 [96.59–131.75]	-0.167	0.449	0.833
Alpha Aminobutyric Acid	21	19.67 [14.64–22.58]	12	21.28 [17.80–25.70]	0.159	0.471	0.835
3 Methylhistidine	21	9.53 [6.30–12.75]	12	10.03 [6.40–22.22]	0.151	0.494	0.838
Phosphoethanolamine	20	2.11 [1.24–3.61]	11	1.83 [1.36–2.08]	-0.145	0.528	0.846
Cystine	21	52.60 [39.64–65.12]	12	57.18 [39.56–67.97]	0.135	0.542	0.846
Taurine	21	158.46 [139.21–189.76]	12	166.79 [149.34–199.33]	0.111	0.618	0.851
Beta Aminoisobutyric Acid	21	1.85 [1.27–2.46]	12	2.17 [1.48–2.96]	0.095	0.665	0.851
Citrulline	21	33.02 [29.37–39.10]	12	31.44 [28.05–34.75]	-0.095	0.671	0.851

Metabolite	n<br>no DM	No diabetes, median [IQR] (μmol/L)	n<br>DM	Diabetes, median [IQR] (μmol/L)	Cliff's δ	Exact p	FDR q
Pipecolic Acid	21	1.35 [1.21–1.63]	12	1.59 [1.11–1.76]	0.095	0.671	0.851
Tryptophane	21	69.78 [64.17–75.22]	12	67.00 [63.00–77.32]	-0.095	0.671	0.851
Beta Alanine	21	2.40 [2.04–3.24]	12	2.18 [1.97–3.33]	-0.087	0.699	0.851
Methionine	21	23.44 [19.96–29.30]	12	25.64 [20.94–28.22]	0.087	0.699	0.851
Asparagine	21	47.26 [41.58–52.99]	12	48.34 [39.30–51.29]	-0.056	0.811	0.930
Ornithine	21	102.64 [89.29–112.01]	12	99.27 [72.97–140.57]	-0.056	0.811	0.930
Phenylalanine	21	73.08 [64.82–80.05]	12	73.92 [66.61–82.61]	0.048	0.839	0.935
Sarcosine	21	1.38 [1.14–2.03]	12	1.51 [0.92–1.86]	-0.040	0.868	0.941
Aspartic Acid	21	30.07 [23.83–36.74]	12	26.81 [22.20–41.81]	-0.008	0.985	1.000
Serine	21	147.51 [140.64–154.93]	12	141.97 [131.98–159.42]	-0.008	0.985	1.000
Arginine	21	85.23 [69.87–98.40]	12	84.84 [77.65–96.98]	-0.000	1.000	1.000
Acetylotyrosine	0	—	0	—	—	—	—
Homocystine	6	0.06 [0.05–0.07]	1	0.06 [0.06–0.06]	—	—	—
Carnosine	0	—	0	—	—	—	—

Metabolite	n no DM	No diabetes, median [IQR] (μmol/L)	n DM	Diabetes, median [IQR] (μmol/L)	Cliff's δ	Exact p	FDR q
Gamma Aminobutyric Acid	0	—	0	—	—	—	—
Saccharopine	0	—	4	0.21 [0.18–0.24]	—	—	—
Anserine	0	—	0	—	—	—	—

The primary CAD cohort was defined as a recorded coronary vessel disease count ≥1 (n = 33). Argininosuccinic acid was available in 26 participants (17 without diabetes and 9 with diabetes) and was the only metabolite that remained significant after FDR correction. Thirty-nine metabolites had valid two-group tests and entered the FDR correction. All 26 argininosuccinic acid estimates were extrapolated below the lowest calibrator (2.64 μmol/L).

Abbreviations: CAD, coronary artery disease; DM, diabetes mellitus; FDR, false discovery rate; IQR, interquartile range; q, false-discovery-rate-adjusted p-value. The table reports Cliff's δ, exact *p*, and FDR *q*.

Table S4. Associations of diabetes with recorded coronary vessel disease and conventional multivessel disease

Outcome	Model	n	OR	95% CI	p-value
Any recorded coronary vessel disease (count ≥1)	Crude	70	1.778	0.633–4.995	0.275
Any recorded coronary vessel disease (count ≥1)	Age + sex	70	1.987	0.685–5.762	0.206
Any recorded coronary vessel disease (count ≥1)	Age + sex + BMI	52	2.281	0.658–7.912	0.194
Any recorded coronary vessel disease (count ≥1)	Age + sex + BMI + recalculated eGFR	52	2.471	0.682–8.955	0.168
Conventional multivessel disease (count ≥2)	Crude	70	1.233	0.391–3.891	0.721
Conventional multivessel disease (count ≥2)	Age + sex	70	1.436	0.433–4.770	0.554

Outcome	Model	n	OR	95% CI	p-value
Conventional multivessel disease (count ≥2)	Age + sex + BMI	52	1.642	0.418–6.457	0.478
Conventional multivessel disease (count ≥2)	Age + sex + BMI + recalculated eGFR	52	1.645	0.405–6.676	0.486

Models including eGFR use the recalculated continuous 2021 CKD-EPI estimate. These disease-status models are secondary analyses and do not establish a CAD-specific metabolomic effect.

Abbreviations: BMI, body mass index; CAD, coronary artery disease; CI, confidence interval; CKD-EPI, Chronic Kidney Disease Epidemiology Collaboration; eGFR, estimated glomerular filtration rate; MVD, multivessel disease; OR, odds ratio.

Table S5. Cohort accounting, model-specific sample sizes, and missingness

Panel A. Source records, participant flow, and model-specific sample sizes

Population or analysis	n
Source spreadsheet records, including repeat records	111
Source records with argininosuccinic acid values	85
Unique participants after consolidation	97
Participants with argininosuccinic acid estimates	73
Participants with recorded coronary vessel count	70
Participants with recorded vessel count 0	37
Participants with recorded vessel count ≥1	33
Participants with recorded vessel count ≥2	18
Participants with vessel count ≥1 and argininosuccinic acid available	26
Participants with vessel count ≥0 and argininosuccinic acid available	52
Disease-restricted cohort complete for age, sex, and BMI	19
Disease-restricted cohort with eGFR ≥60 sensitivity analysis	20
Disease-restricted cohort after exclusion of two highest ASA values	24

Panel B. Variable-level missingness in the primary CAD cohort (n = 33)

Variable	Available n	Missing n
Argininosuccinic acid	26	7
Age	33	0
Sex	33	0
BMI	25	8
Recalculated 2021 CKD-EPI eGFR	33	0
Creatinine	33	0

Panel C. Argininosuccinic acid availability by diabetes status

Group	Group n	ASA available n	ASA missing n	ASA missing (%)
Diabetes	12	9	3	25.0%
No diabetes	21	17	4	19.0%

The reasons for the seven unavailable argininosuccinic acid estimates in the disease-restricted cohort are unknown in the available analytic records (4/21 without diabetes; 3/12 with diabetes). Missing values were not imputed; possible selection related to sample quality or concentration cannot be excluded. All available argininosuccinic acid estimates were extrapolated below the lowest calibrator (2.64 μmol/L).

Abbreviations: ASA, argininosuccinic acid; BMI, body mass index; CAD, coronary artery disease; CKD-EPI, Chronic Kidney Disease Epidemiology Collaboration; eGFR, estimated glomerular filtration rate; MVD, multivessel disease.

Table S6. Renal-function analyses for argininosuccinic acid in the primary CAD cohort

Panel A. Renal-function distributions among participants with argininosuccinic acid available

Group	n	Recalculated eGFR, median [IQR] (mL/min/1.73 m²)	Creatinine, median [IQR] (mg/dL)
Diabetes	9	55.50 [49.40–91.70]	1.15 [0.94–1.37]
No diabetes	17	87.40 [76.80–97.50]	0.84 [0.73–0.96]

Panel B. Spearman correlations

Analysis	n	Spearman ρ	p-value
Pooled: ASA vs recalculated eGFR	26	-0.445	0.023
Pooled: ASA vs creatinine	26	0.486	0.012
No diabetes: ASA vs recalculated eGFR	17	-0.433	0.082
Diabetes: ASA vs recalculated eGFR	9	-0.083	0.831
No diabetes: ASA vs creatinine	17	0.394	0.118
Diabetes: ASA vs creatinine	9	0.117	0.765

Panel C. DM-adjusted renal models with HC3 robust standard errors

Model	Term	n	Estimate	95% CI	p-value
ASA z score ~ DM + recalculated eGFR	Diabetes	26	1.159	-0.027–2.346	0.055
ASA z score ~ DM + recalculated eGFR	Recalculated eGFR	26	-0.002	-0.023–0.019	0.841

Model	Term	n	Estimate	95% CI	p-value
ASA z score ~ DM + creatinine	Diabetes	26	1.172	-0.045–2.389	0.058
ASA z score ~ DM + creatinine	Creatinine	26	0.109	-1.222–1.441	0.867

Argininosuccinic acid was standardized once across the 26 available primary-cohort observations before model fitting. The pooled renal correlations indicate a renal contribution, whereas the small stratified and DM-adjusted analyses are imprecise and do not establish renal independence.

Abbreviations: ASA, argininosuccinic acid; CAD, coronary artery disease; CI, confidence interval; CKD-EPI, Chronic Kidney Disease Epidemiology Collaboration; DM, diabetes mellitus; eGFR, estimated glomerular filtration rate; HC3, heteroskedasticity-consistent covariance estimator type 3; IQR, interquartile range; ϱ, Spearman's rank correlation coefficient.

Table S7. Leave-one-out influence analysis for the age- and sex-adjusted argininosuccinic acid model

Omitted observation index	n	Diabetes coefficient	95% CI	HC3 p-value
1	25	0.877	-0.063–1.817	0.066
2	25	1.228	0.130–2.327	0.030
3	25	1.238	0.023–2.453	0.046
4	25	1.201	0.106–2.296	0.033
5	25	0.886	-0.028–1.801	0.057
6	25	1.164	-0.103–2.430	0.070
7	25	1.179	-0.112–2.469	0.071
8	25	1.362	0.171–2.553	0.027
9	25	1.211	0.127–2.294	0.030
10	25	1.168	0.075–2.262	0.037
11	25	1.170	0.084–2.256	0.036
12	25	1.159	0.068–2.251	0.038
13	25	1.197	0.114–2.280	0.032
14	25	1.169	0.084–2.255	0.036
15	25	1.174	0.081–2.267	0.037
16	25	1.163	0.077–2.249	0.037
17	25	1.293	0.114–2.471	0.033
18	25	1.169	0.080–2.258	0.037
19	25	1.181	0.084–2.279	0.036
20	25	1.172	0.079–2.264	0.037

Omitted observation index	n	Diabetes coefficient	95% CI	HC3 p-value
21	25	1.175	0.082–2.269	0.036
22	25	1.167	0.075–2.259	0.037
23	25	1.293	0.102–2.484	0.035
24	25	1.344	0.146–2.543	0.030
25	25	1.192	0.093–2.290	0.035
26	25	1.199	0.094–2.304	0.035

Observation indices correspond to the order of the complete-case argininosuccinic acid analysis dataset and are not patient identifiers. Several leave-one-out HC3 confidence intervals crossed zero, demonstrating sensitivity of precision to individual observations despite a consistently positive coefficient.

Abbreviations: CI, confidence interval; HC3, heteroskedasticity-consistent covariance estimator type 3.

Table S8. Formal diabetes-by-CAD interaction analyses for argininosuccinic acid

Model	n	Interaction term	Estimate	95% CI	p-value	SE method
Crude DM x CAD interaction	52	DM:CAD	1.046	-0.454–2.546	0.167	HC3 robust
Age + sex adjusted DM x CAD interaction	52	DM:CAD	1.056	-0.517–2.629	0.183	HC3 robust
Log outcome, age + sex adjusted DM x CAD interaction	52	DM:CAD	0.962	-0.561–2.485	0.210	HC3 robust

The interaction term was not statistically significant in any model; therefore, these data do not establish that the diabetes-associated argininosuccinic acid signal is specific to recorded CAD.

Abbreviations: CAD, coronary artery disease; CI, confidence interval; DM, diabetes mellitus; HC3, heteroskedasticity-consistent covariance estimator type 3; SE, standard error.

Table S9. Assay performance and analyte-level data quality.

Analyte	Calibration range (μmol/L)	Calibration curve	r^2	LOD (μmol/L)	LOQ S/N=10 (μmol/L)	Calibrator accuracy % (Cal1/2/3)	Control accuracy % (I/II/III)	Level II CV % (n=6)
1-Metylhistidine	1.39–19.7	0.2165x -0.0188	0.99439	0.0049	0.01633	107/90/103	96/102/94	7.2
3-Methylhistidine	3.95–123	0.01682x -0.00497	0.99910	0.1561	0.5204	105/94/101	104/107/104	3.9
4-Hydroxyproline	4.8–217	0.02848x -0.00506	0.99960	0.05809	0.1936	105/95/100	95/98/93	2.7
Acetylotyrosine	2.34–97.7	0.1155x +0.00393	0.99876	0.01084	0.03612	106/93/101	93/96/92	3.0

Analyte	Calibration range (μmol/L)	Calibration curve	r²	LOD (μmol/L)	LOQ S/N=10 (μmol/L)	Calibrator accuracy % (Cal1/2/3)	Control accuracy % (I/II/III)	Level II CV % (n=6)
Adenosylhomocysteine	1.52–49.7	0.1072x -0.00598	0.99894	0.2214	0.7379	105/94/101	87/95/90	2.7
Alanine	106–1381	0.0003567x -3.92e-05	0.99722	4.84	16.13	106/92/102	95/96/93	1.4
Allo-Isoleucine	1.78–208	0.4447x +0.0121	0.99976	0.07335	0.2445	104/96/100	93/102/94	1.9
alpha-Aminoadipic acid	2.9–23.5	0.1182x -0.00112	0.99865	0.00846	0.0282	103/95/101	93/97/94	2.9
alpha-Aminobutyric acid	2.78–116	0.03686x -0.00844	0.99966	0.07794	0.2598	104/95/100	96/101/95	1.0
Anserine	2.2–19.2	0.07697x +0.0179	0.99657	0.01768	0.05893	105/93/102	95/95/86	6.2
Arginine	6.66–369	0.01143x -0.0106	0.99869	0.1821	0.6071	105/93/102	96/94/91	4.8
Argininosuccinic acid	2.64–187	0.1323x -0.00995	0.99951	0.0221	0.07368	106/94/101	100/101/94	7.0
Asparagine	13.2–285	0.02355x -0.0178	0.99681	0.5211	1.737	106/91/103	94/99/94	3.4
Aspartic acid	13.1–293	0.006323x -0.00647	0.99973	1.156	3.853	102/97/101	104/101/95	1.1
beta-Alanine	5.4–121	0.07269x -0.00249	0.99994	0.03505	0.1168	99/101/100	99/103/96	5.9
beta-Aminoisobutyric acid	2.39–50.3	0.06052x +0.00495	0.99843	0.09547	0.3182	105/93/101	95/96/90	2.1
Carnosine	4.91–50.4	0.08954x +0.0442	0.99758	0.1954	0.6512	106/93/101	99/103/94	4.0
Citrulline	7.76–241	0.05338x -0.0526	0.99917	0.1795	0.5983	106/94/101	100/99/93	1.4
Cystathionine	2.89–57.1	0.5409x -0.358	0.99972	0.008122	0.02707	102/97/101	100/97/89	7.1
Cysteine sulfate	7.8–59.6	0.03002x -0.0382	0.99847	0.2696	0.8986	102/96/102	97/99/93	2.5
Cystine	3.53–186	0.02416x +0.00119	0.99939	0.1047	0.3492	106/93/101	95/105/96	5.9
Ethanolamine	7.61–283	0.02042x -0.0109	0.99955	2.154	7.179	105/94/100	99/100/96	3.1
gamma-Aminobutyric acid	2.62–19.3	0.04496x -0.0356	0.99392	0.2375	0.7915	106/91/103	97/99/93	2.8
Glutamic acid	31.4–938	0.001235x +0.00638	0.99979	0.2711	0.9036	103/97/100	102/103/96	3.9
Glutamine	172–1902	0.004282x +0.0809	0.99706	0.9814	3.271	104/94/103	96/96/95	9.3
Glycine	141–1425	0.001231x -0.00398	0.99848	1.182	3.939	104/95/101	97/98/94	0.7

Analyte	Calibration range (µmol/L)	Calibration curve	r²	LOD (µmol/L)	LOQ S/N=10 (µmol/L)	Calibrator accuracy % (Cal1/2/3)	Control accuracy % (I/II/III)	Level II CV % (n=6)
Histidine	22.4–352	0.004664x +0.00252	0.99975	0.56	1.867	102/98/101	99/100/97	3.4
Homocitrulline	4.83–57.6	0.1676x -0.075	0.99837	0.4514	1.505	103/95/102	93/100/94	3.2
Homocystine	1.75–28.7	0.1251x -0.00243	0.99791	0.1322	0.4408	105/93/102	97/105/92	2.3
Hydroxylysine	2.29–39.2	0.12x +0.0462	0.99956	-	-	102/97/101	102/103/98	1.7
Lysine	18.5–746	0.3334x -0.3	0.99818	2.229	7.43	109/90/101	95/94/90	4.0
Methionine	5.67–203	0.05815x +0.00104	0.99920	0.009686	0.03229	106/94/101	98/104/98	2.6
Ornithine	11.2–498	0.001197x - 0.000589	0.99810	1.706	5.685	107/91/102	105/102/94	3.5
Phenylalanine	24.9–769	0.01961x -0.00803	0.99918	0.4399	1.466	103/95/101	100/103/97	2.1
Phosphoethanolamine	4.7–167	0.1026x +0.0962	0.99902	0.1117	0.3724	108/92/101	99/106/96	5.8
Pipecolic acid	1.76–40.6	1.027x -0.0256	0.99942	0.2933	0.9778	103/96/101	97/104/98	2.2
Proline	39.7–770	0.001367x +0.00302	0.99982	0.3554	1.185	101/98/101	98/101/94	4.0
Saccharopine	2.34–18.6	0.2387x -0.0249	0.99599	0.01087	0.03624	104/93/103	107/112/109	4.6
Sarcosine	2.38–39.2	0.1006x +0.000609	0.99927	0.1445	0.4818	103/96/101	98/103/97	1.9
Serine	59.4–755	0.01342x -0.0365	0.99908	0.6433	2.144	103/96/101	102/99/95	1.5
Taurine	9.91–534	0.005492x - 0.000314	0.99977	0.3043	1.014	102/97/101	98/101/96	0.6
Threonine	29.6–586	0.03261x -0.0558	0.99927	0.2861	0.9536	104/95/101	97/99/93	3.1
Tryptophane	9.69–274	0.01187x -0.00923	1.00000	0.8809	2.936	100/100/100	94/103/92	7.1
Tyrosine	19–375	0.02072x -0.00326	0.99936	0.3276	1.092	103/96/101	104/106/97	1.0
Valine	47.8–719	0.001567x - 0.000245	0.99877	1.046	3.487	104/94/101	99/101/96	1.9

LOD, limit of detection; LOQ, limit of quantification; S/N, signal-to-noise ratio; CV, coefficient of variation. Calibration used the three levels of the 3PLUS1 plasma calibrator set. Because the manufacturer does not declare limits of detection, LOD and LOQ were estimated from the signal-to-noise ratio of the lowest calibrator as the concentrations corresponding to S/N = 3 and S/N = 10, assuming a proportional response and constant noise below Cal1; Calibrator accuracy is the back-calculated concentration as a percentage of nominal at each level; control accuracy is the corresponding value for the three MassCheck control levels; the Level II CV is the coefficient of variation of the six control determinations distributed in the reported quality-control series. The estimated LOQ does not validate quantitative accuracy below the lowest calibrator.

Abbreviations: CAD, collision gas; CE, collision energy; CV, coefficient of variation; CXP, collision-cell exit potential; DP, declustering potential; EP, entrance potential; GS1, nebulizer gas; GS2, heater gas; IS, stable-isotope-labeled internal standard; LOD, limit of detection; LOQ, limit of quantification; MRM, multiple-reaction monitoring; Q1, precursor ion; Q3, product ion; RT, retention time; S/N, signal-to-noise ratio.

Table S10. Chromatographic solvent and flow-rate program.

Time (min)	Mobile phase B (%)	Mobile phase A (%)	Flow rate (mL/min)
0.0	0	100	0.8
0.5	0	100	0.8
1.0	11	89	0.8
6.3	11	89	0.8
12.3	20	80	0.8
14.8	40	60	0.8
14.9	100	0	0.8
16.4	100	0	0.8
16.5	100	0	0.3
17.4	100	0	0.3
17.5	100	0	0.8
17.7	0	100	0.8
19.3	0	100	0.8
19.8	0	100	1.8
20.4	0	100	1.8
20.5	0	100	0.8

Column: Chromsystems MassChrom analytical column for amino-acid analysis (75100), 25 °C. Injection volume 5 μL; 3 s standard needle wash at the flush port. Upper pressure limit 280 bar; total run time 20.5 min. A Valco switching valve diverted the column effluent to waste between 19.8 and 20.4 min. Mobile phase A, Chromsystems 75001; mobile phase B, Chromsystems 75002.

Table S11. Scheduled multiple-reaction-monitoring transitions and compound-dependent parameters.

Compound	Q1 (m/z)	Q3 (m/z)	RT (min)	DP (V)	EP (V)	CE (V)	CXP (V)	Role
1-Metylhistidine	170.1	81.1	14.9	80	10	55	20	Quantifier
1-Metylhistidine	170.1	83	14.9	80	10	35	25	Qualifier
1-Metylhistidine (IS)	173	81.1	14.9	20	10	55	20	Internal standard
3-Methylhistidine	171.1	97.1	15.4	90	10	27	14	Quantifier
3-Methylhistidine	171.1	110.1	15.4	90	10	22	20	Qualifier
3-Methylhistidine (IS)	173.1	112.1	15.4	95	10	23	15	Internal standard
4-Hydroxyproline	132	86.1	13.4	45	10	20	20	Quantifier
4-Hydroxyproline	132	68	13.4	45	10	30	13	Qualifier

Compound	Q1 (m/z)	Q3 (m/z)	RT (min)	DP (V)	EP (V)	CE (V)	CXP (V)	Role
4-Hydroxyproline (IS)	135	89	13.4	35	10	20	20	Internal standard
Acetylotyrosine	224	136.1	3.05	76	10	27	17	Quantifier
Acetylotyrosine	224	178.1	3.05	76	10	14	26	Qualifier
Acetylotyrosine (IS)	227	137.1	3.05	55	10	27	14	Internal standard
Adenosylhomocysteine	385	136	14.8	100	10	25	16	Quantifier
Adenosylhomocysteine	385	88	14.8	100	10	67	20	Qualifier
Adenosylhomocysteine (IS)	389	138	14.8	50	10	27	17	Internal standard
Alanine	91	45	10.7	61	10	20	12	Quantifier
Alanine	91	73	10.7	61	10	12	17	Qualifier
Alanine (IS)	94	48.1	10.7	50	10	20	13	Internal standard
Allo-Isoleucine	132.1	69.101	6.7	70	12	25	25	Quantifier
Allo-Isoleucine	132.1	44.101	6.7	70	12	32	20	Qualifier
Allo-Isoleucine (IS)	142.1	78	6.7	18	8	26	20	Internal standard
alpha-Aminoadipic acid	162.1	98	11	15	10	22	20	Quantifier
alpha-Aminoadipic acid	162.1	116.1	11	15	10	17	20	Qualifier
alpha-Aminoadipic acid (IS)	165	101.1	11	15	10	23	11	Internal standard
alpha-Aminobutyric acid	104.1	58.1	9.2	50	10	15	15	Quantifier
alpha-Aminobutyric acid	104.1	41	9.2	50	10	38	13	Qualifier
alpha-Aminobutyric acid (IS)	107	61.1	9.2	60	10	15	15	Internal standard
Anserine	241.1	96.1	15.7	70	10	42	10	Quantifier
Anserine	241.1	68.1	15.7	70	10	61	7	Qualifier
Anserine (IS)	245.1	109.1	15.7	70	10	30	12	Internal standard
Arginine	176.1	71.1	13.7	95	10	30	11	Quantifier
Arginine	176.1	60.1	13.7	95	10	20	16	Qualifier
Arginine (IS)	181	74.1	13.7	20	10	33	20	Internal standard
Argininosuccinic acid	291	70.1	17.8	90	10	56	15	Quantifier
Argininosuccinic acid	291	116.1	17.8	90	10	27	20	Qualifier
Argininosuccinic acid (IS)	301	75	17.8	20	10	50	20	Internal standard
Asparagine	133	74	14	17	10	20	20	Quantifier
Asparagine	133	87.1	14	17	10	15	20	Qualifier
Asparagine (IS)	138	76	14	67	10	20	20	Internal standard
Aspartic acid	134	74	15.7	64	10	17	19	Quantifier
Aspartic acid	134	88	15.7	64	10	14	14	Qualifier
Aspartic acid (IS)	137	75	15.7	60	10	22	14	Internal standard

Compound	Q1 (m/z)	Q3 (m/z)	RT (min)	DP (V)	EP (V)	CE (V)	CXP (V)	Role
beta-Alanine	90.1	72	5.8	65	10	11	10	Quantifier
beta-Alanine	90.1	30	5.8	65	10	23	10	Qualifier
beta-Alanine (IS)	94	32.1	5.8	65	10	25	15	Internal standard
beta-Aminoisobutyric acid	104	86	4.9	64	10	11	10	Quantifier
beta-Aminoisobutyric acid	104	30.1	4.9	64	10	26	13	Qualifier
beta-Aminoisobutyric acid (IS)	107	89.1	4.9	55	10	13	16	Internal standard
Carnosine	227.1	210.1	14.8	90	10	16	12	Quantifier
Carnosine	227.1	156.1	14.8	90	10	20	15	Qualifier
Carnosine (IS)	231	156.1	14.8	30	10	22	20	Internal standard
Citrulline	176	159.1	15.6	20	10	15	20	Quantifier
Citrulline	176	70	15.6	20	10	30	16	Qualifier
Citrulline (IS)	180.1	74	15.6	30	10	35	20	Internal standard
Cystathionine	223	134	17.8	80	10	19	15	Quantifier
Cystathionine	223	88	17.8	80	10	40	23	Qualifier
Cystathionine (IS)	227.1	92	17.8	120	10	40	25	Internal standard
Cysteine sulfate	202	120	18.9	91	10	20	20	Quantifier
Cysteine sulfate	202	74	18.9	91	10	35	20	Qualifier
Cysteine sulfate (IS)	205.1	123	18.9	40	10	20	15	Internal standard
Cystine	241	152	17.8	65	10	19	25	Quantifier
Cystine	241	120	17.8	65	10	27	14	Qualifier
Cystine (IS)	245.1	154	17.8	62	10	19	10	Internal standard
Ethanolamine	62	44.1	4.5	60	10	15	11	Quantifier
Ethanolamine (IS)	66.1	48.1	4.5	80	10	14	13	Internal standard
gamma-Aminobutyric acid	104	87	4.25	50	10	15	10	Quantifier
gamma-Aminobutyric acid	104	69	4.25	50	10	20	10	Qualifier
gamma-Aminobutyric acid (IS)	110	93	4.25	59	10	15	10	Internal standard
Glutamic acid	149.1	85.1	12.7	70	10	24	20	Quantifier
Glutamic acid	149.1	131.1	12.7	70	10	12	15	Qualifier
Glutamic acid (IS)	151.1	133.1	12.7	72	10	14	17	Internal standard
Glutamine	148	131	14.2	50	10	14	16	Quantifier
Glutamine	148	85.1	14.2	50	10	24	20	Qualifier
Glutamine (IS)	154.1	136.1	14.2	85	9	10	24	Internal standard
Glycine	75.9	30	12.4	65	10	20	8	Quantifier
Glycine	75.9	48	12.4	65	10	10	14	Qualifier

Compound	Q1 (m/z)	Q3 (m/z)	RT (min)	DP (V)	EP (V)	CE (V)	CXP (V)	Role
Glycine (IS)	79	32	12.4	64	10	23	10	Internal standard
Histidine	157	84	14.7	20	10	35	23	Quantifier
Histidine	157	94	14.7	20	10	35	23	Qualifier
Histidine (IS)	159	85	14.7	95	11	34	21	Internal standard
Homocitrulline	190.1	173.1	14.8	55	10	15	20	Quantifier
Homocitrulline	190.1	84.1	14.8	55	10	30	10	Qualifier
Homocitrulline (IS)	193	176.1	14.8	12	10	16	20	Internal standard
Homocystine	269	136	16.6	63	10	15	18	Quantifier
Homocystine	269	88	16.6	63	10	49	23	Qualifier
Homocystine (IS)	277	140	16.6	15	10	17	18	Internal standard
Hydroxylysine	163	128.1	16.1	70	10	16	16	Quantifier
Hydroxylysine	163	82	16.1	70	10	25	20	Qualifier
Hydroxylysine (IS)	168.1	87.1	16.1	50	10	25	16	Internal standard
Isoleucine	132.1	69.1	6.4	65	10	25	17	Quantifier
Isoleucine	132.1	41.1	6.4	65	10	37	19	Qualifier
Isoleucine (IS)	139.1	74.1	6.4	65	10	25	14	Internal standard
Leucine	132.1	44	6.3	70	10	32	15	Quantifier
Leucine	132.1	43	6.3	70	10	36	13	Qualifier
Leucine (IS)	135.1	46.1	6.3	70	10	35	13	Internal standard
Lysine	147	84.1	15	60	10	22	20	Quantifier
Lysine	147	56	15	60	10	41	15	Qualifier
Lysine (IS)	149.9	59	15	16	10	45	17	Internal standard
Methionine	150	56	7.3	65	10	23	15	Quantifier
Methionine	150	61	7.3	65	10	30	17	Qualifier
Methionine (IS)	153	64	7.3	70	10	33	13	Internal standard
Ornithine	134.1	71.1	15.8	45	10	24	18	Quantifier
Ornithine	134.1	117	15.8	50	10	13	14	Qualifier
Ornithine (IS)	139.1	76.1	15.8	50	10	26	15	Internal standard
Phenylalanine	167.1	104.1	6.3	65	10	40	18	Quantifier
Phenylalanine	167.1	77	6.3	65	10	54	21	Qualifier
Phenylalanine (IS)	171	81.1	6.3	70	10	54	15	Internal standard
Phosphoethanolamine	142	44	17.7	15	10	30	16	Quantifier
Phosphoethanolamine (IS)	146	48	17.7	15	10	25	25	Internal standard
Phosphoserine	186	88	18.5	45	10	16	10	Quantifier

Compound	Q1 (m/z)	Q3 (m/z)	RT (min)	DP (V)	EP (V)	CE (V)	CXP (V)	Role
Phosphoserine	186	70	18.5	45	10	29	10	Qualifier
Phosphoserine (IS)	190.1	92	18.5	50	8	16	10	Internal standard
Pipecolic acid	130	84.1	10.4	80	10	23	14	Quantifier
Pipecolic acid	130	56	10.4	80	10	40	15	Qualifier
Pipecolic acid (IS)	139.1	61.1	10.4	70	10	40	15	Internal standard
Proline	117.1	71	12.1	70	10	25	18	Quantifier
Proline	117.1	43	12.1	46	10	37	12	Qualifier
Proline (IS)	123	77.1	12.1	65	10	24	12	Internal standard
Saccharopine	277	84.1	18	100	10	30	21	Quantifier
Saccharopine	277	213.1	18	100	10	20	11	Qualifier
Saccharopine (IS)	281	88.1	18	45	10	37	23	Internal standard
Sarcosine	90	44	12.5	64	10	16	11	Quantifier
Sarcosine (IS)	93	47	12.5	65	10	17	15	Internal standard
Serine	106	60	13.2	60	10	15	15	Quantifier
Serine	106	42	13.2	60	10	30	11	Qualifier
Serine (IS)	109	63.1	13.2	65	10	17	15	Internal standard
Taurine	126	44	8.8	95	10	30	12	Quantifier
Taurine	126	30	8.8	95	10	30	15	Qualifier
Taurine (IS)	130	48.1	8.8	96	12	33	13	Internal standard
Threonine	120.1	56	11.9	50	10	23	15	Quantifier
Threonine	120.1	102	11.9	50	10	14	12	Qualifier
Threonine (IS)	125	107.1	11.9	70	10	13	15	Internal standard
Tryptophane	206.1	147.1	5.9	65	10	26	20	Quantifier
Tryptophane	206.1	119.1	5.9	65	10	37	23	Qualifier
Tryptophane (IS)	210.1	122.1	5.9	60	10	38	20	Internal standard
Tyrosine	183.1	166.1	7.4	80	10	15	16	Quantifier
Tyrosine	183.1	137.1	7.4	80	10	17	13	Qualifier
Tyrosine (IS)	186	140	7.4	65	10	12	15	Internal standard
Valine	119.1	73.1	7.75	20	10	15	15	Quantifier
Valine	119.1	55	7.75	20	10	26	15	Qualifier
Valine (IS)	126.1	80.1	7.75	70	10	17	17	Internal standard

Q1, precursor ion; Q3, product ion; RT, expected retention time; DP, declustering potential; EP, entrance potential; CE, collision energy; CXP, collision-cell exit potential; IS, stable-isotope-labelled internal standard. All transitions were acquired in positive-ion mode with Q1 and Q3 at unit resolution. Source parameters: ion-spray voltage 5000 V, source temperature 550 °C, curtain gas 35 psi, nebulizer gas (GS1) 35 psi, heater gas (GS2) 40 psi, collision gas (CAD) setting 7. Dwell times were computed automatically by the scheduled-MRM algorithm within a permitted range of 3–250 ms (effective range 5.67–79.90 ms). A total of 141 transitions covering 48 analytes and 48 internal standards were monitored. Compound names are reproduced verbatim from the acquisition method and may differ in spelling from the standardised names used in the main text and in Table S3;

specifically, “1-Metylhistidine” corresponds to 1-methylhistidine, “Tryptophane” to tryptophan, “Acetylotyrosine” to N-acetyltyrosine and “Cysteine sulfate” to cysteine-S-sulfate. Leucine, isoleucine, and phosphoserine occur in this 48-analyte acquisition method but are absent as participant-level concentration columns from the 45-analyte statistical database and were therefore excluded from both screens (39 valid tests in the CAD cohort; 41 in the full cohort).

Figure S1. Recorded coronary vessel disease by diabetes status

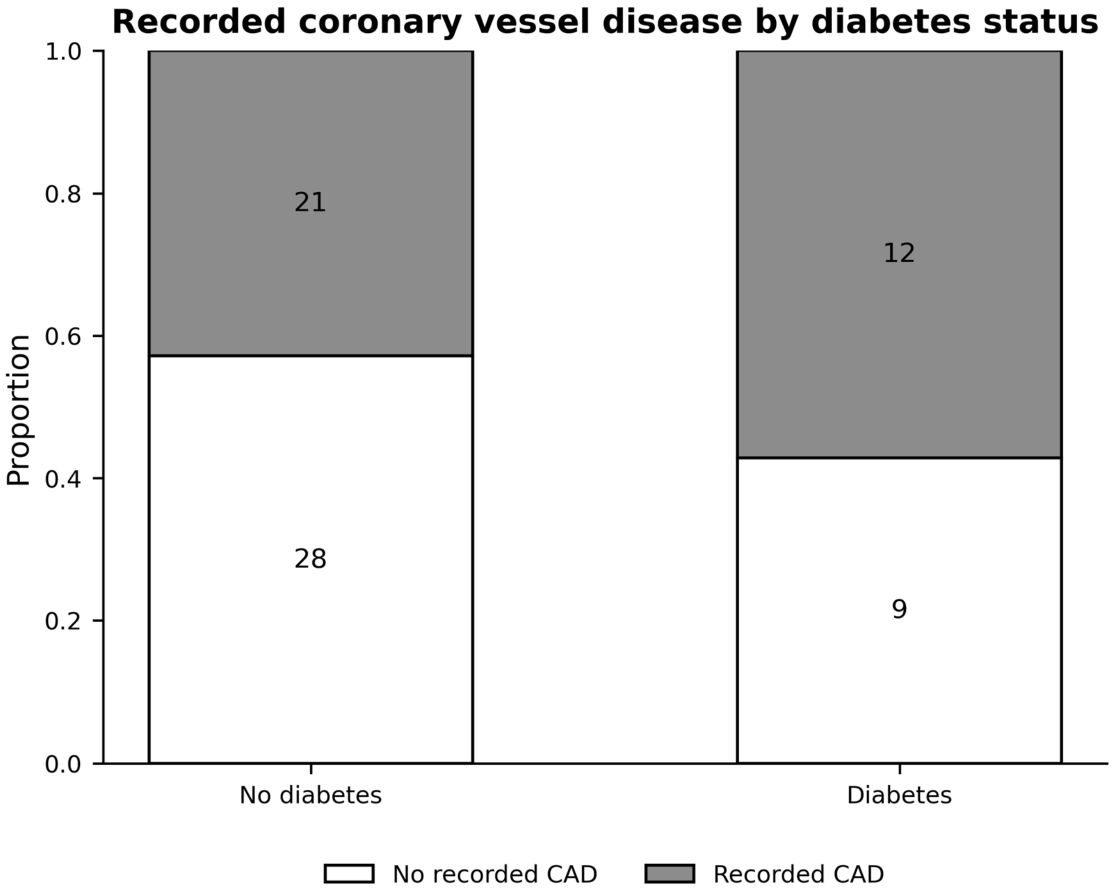

Proportions are shown in the angiography-evaluable cohort (n = 70). Recorded coronary vessel disease was defined as vessel count ≥1. Counts are displayed within each bar.

Abbreviations: CAD, coronary artery disease; DM, diabetes mellitus.

Figure S2. Amino-acid effect sizes in the full metabolic cohort

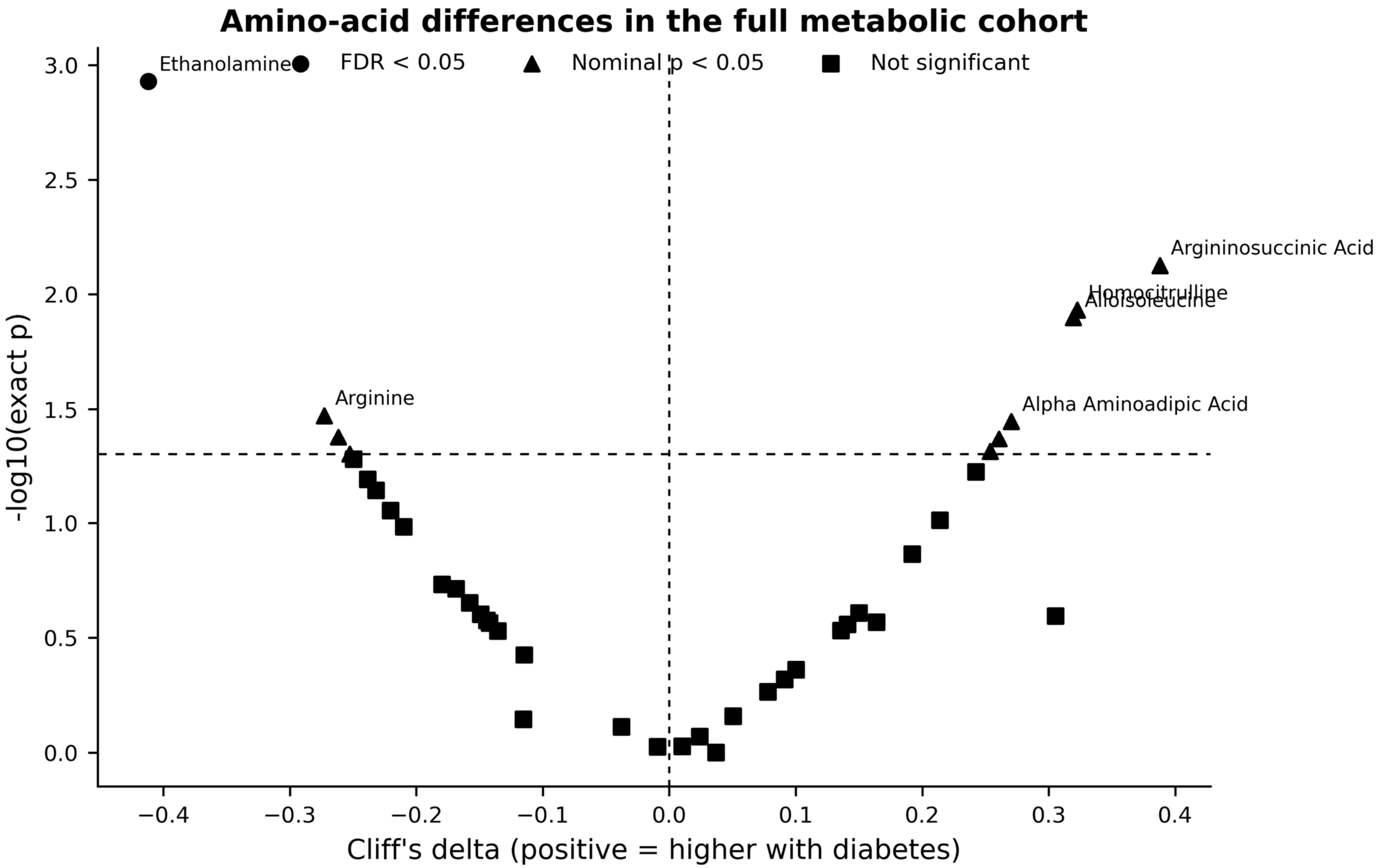

Cliff’s delta is positive when concentrations are higher in participants with diabetes. Symbols distinguish the single FDR-significant finding (ethanolamine), nominal associations, and nonsignificant comparisons. This analysis provides full-cohort context and is not evidence of CAD specificity. The FDR family contained 41 valid comparisons; argininosuccinic acid estimates were extrapolated below the calibration range.

Abbreviations: CAD, coronary artery disease; DM, diabetes mellitus; FDR, false discovery rate.

Figure S3. Cohort derivation and model-specific sample sizes

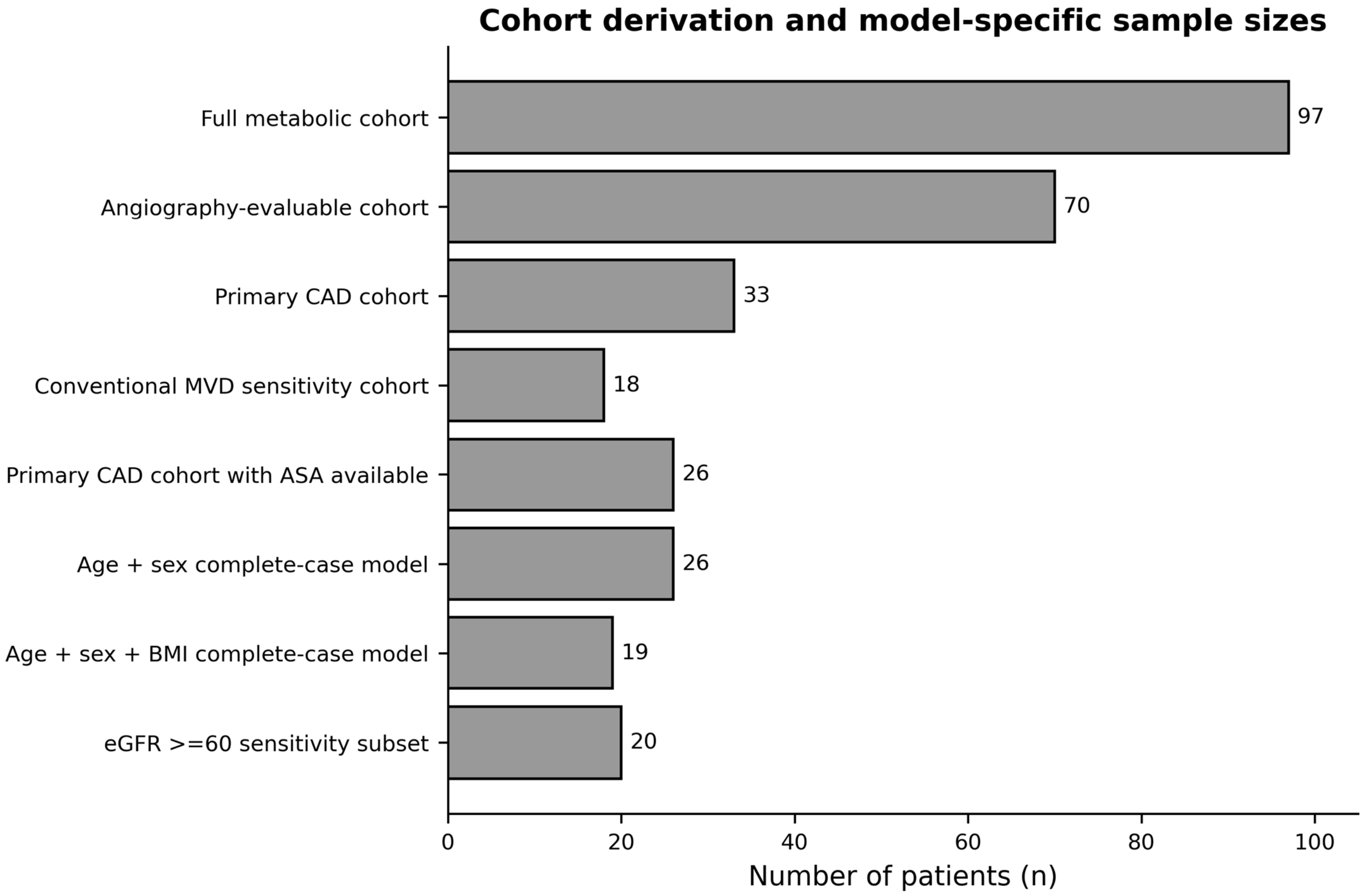

Bars summarize the full metabolic cohort, the angiography-evaluable cohort, the disease-restricted cohorts, and the principal model/sensitivity-analysis sample sizes. The 111 raw spreadsheet records include repeats that were consolidated into 97 participants; 85 source-level argininosuccinic acid values correspond to 73 participant-level estimates.

Abbreviations: ASA, argininosuccinic acid; CAD, coronary artery disease; DM, diabetes mellitus; eGFR, estimated glomerular filtration rate; MVD, multivessel disease.

Figure S4. Leave-one-out influence analysis

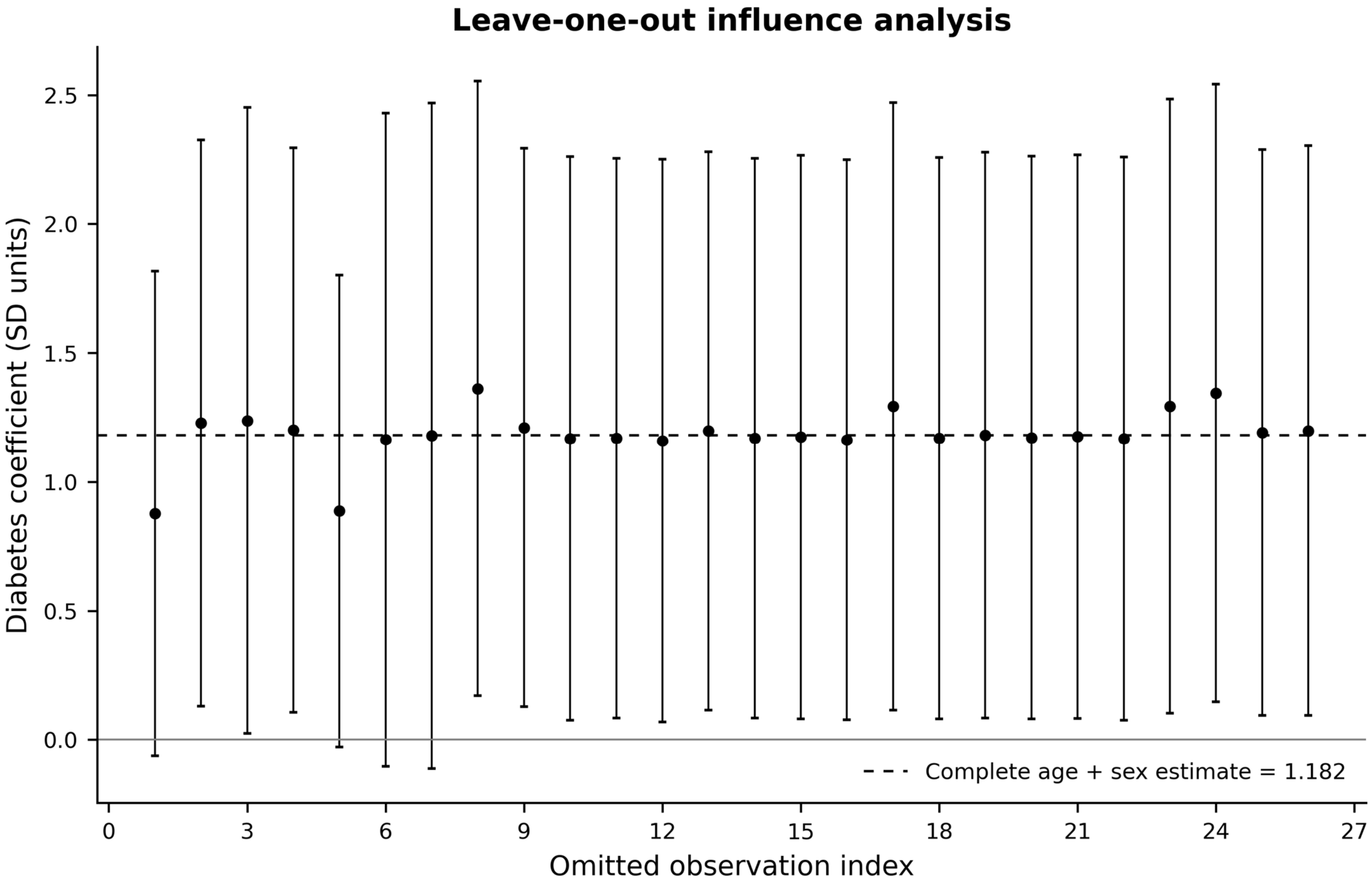

Each point shows the age- and sex-adjusted diabetes coefficient after omitting one observation; vertical bars indicate HC3-robust 95% confidence intervals. The dashed line indicates the complete age- and sex-adjusted coefficient.

Abbreviations: CI, confidence interval; DM, diabetes mellitus; HC3, heteroskedasticity-consistent covariance estimator type 3.

Figure S5. Argininosuccinic acid and recalculated renal function

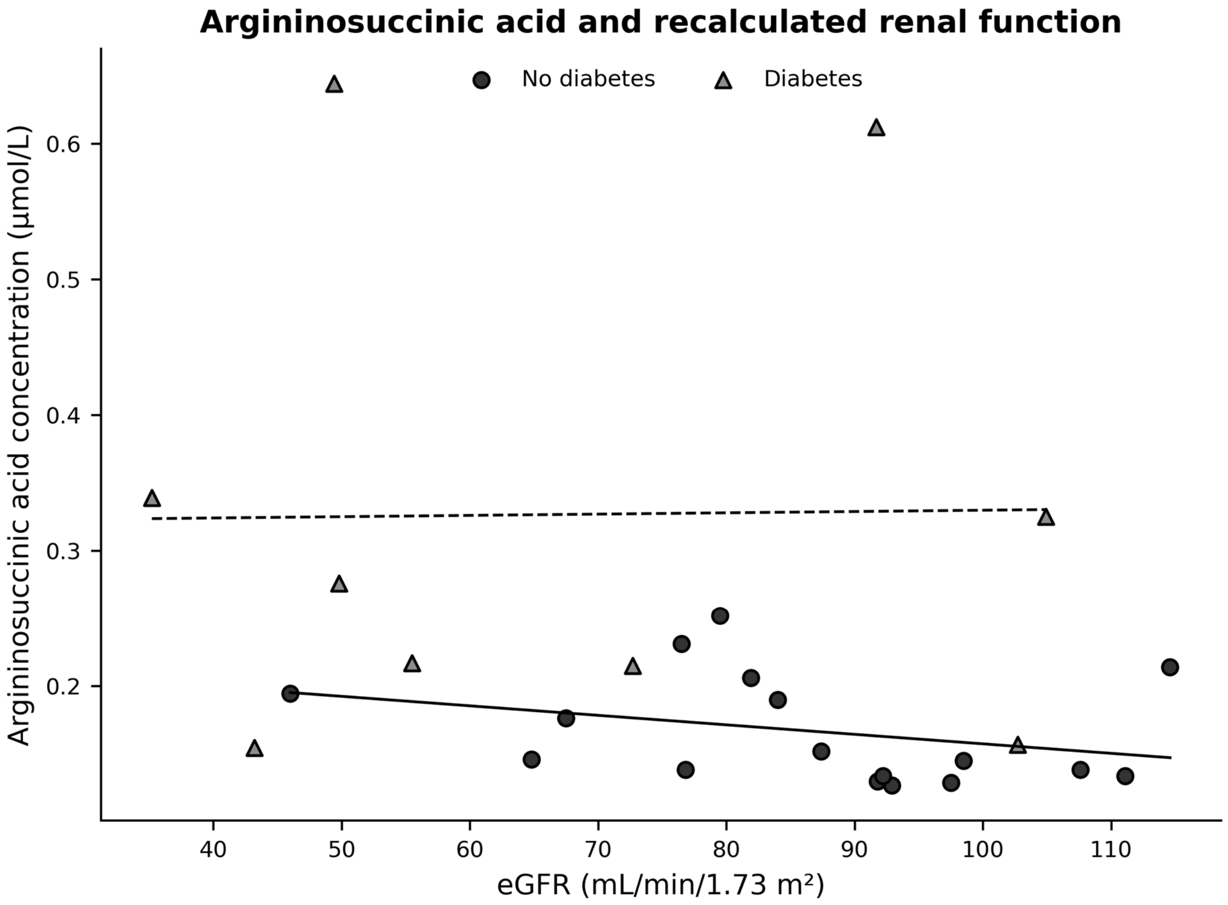

Scatterplot of estimated argininosuccinic acid concentration and continuous 2021 CKD-EPI eGFR in the primary CAD cohort, stratified by diabetes status. Pooled Spearman $\varrho = -0.445$ ($p = 0.023$); within-group correlations were not statistically significant. All plotted values are extrapolated below the lowest calibrator and are semiquantitative.

Abbreviations: ASA, argininosuccinic acid; CAD, coronary artery disease; CKD-EPI, Chronic Kidney Disease Epidemiology Collaboration; DM, diabetes mellitus; eGFR, estimated glomerular filtration rate; ϱ, Spearman's rank correlation coefficient.

Figure S6. Argininosuccinic acid by diabetes and recorded CAD status

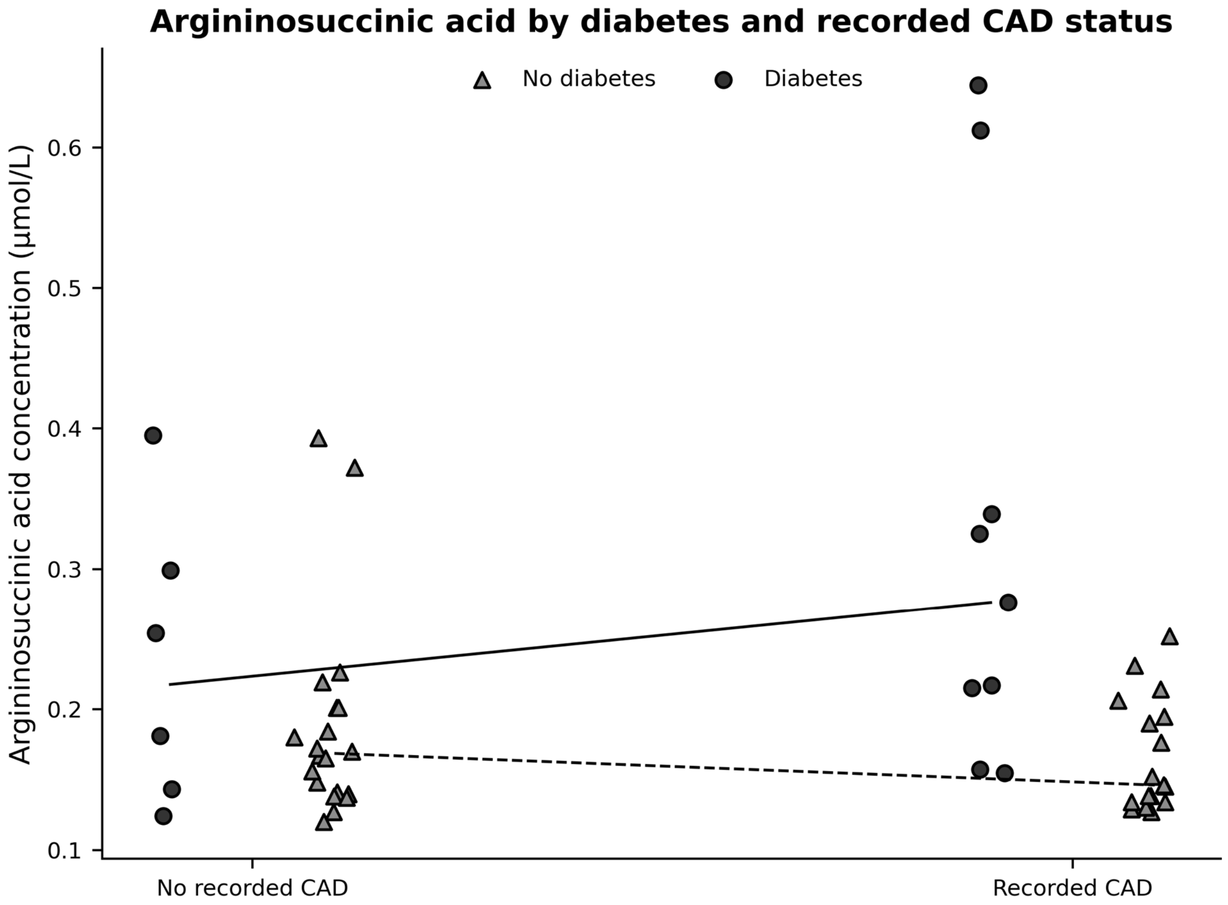

Estimated argininosuccinic acid concentrations are shown by diabetes status and recorded CAD status among participants with available angiographic and metabolite data. Lines connect group medians. Formal diabetes-by-CAD interaction testing was not statistically significant. All estimates are extrapolated below the lowest calibrator.

Abbreviations: ASA, argininosuccinic acid; CAD, coronary artery disease; DM, diabetes mellitus.
